# Genomic, Physiologic, and Symbiotic Characterization of *Serratia marcescens* Strains Isolated from the Mosquito *Anopheles stephensi*

**DOI:** 10.3389/fmicb.2017.01483

**Published:** 2017-08-10

**Authors:** Shicheng Chen, Jochen Blom, Edward D. Walker

**Affiliations:** ^1^Department of Microbiology and Molecular Genetics, Michigan State University East Lansing, MI, United States; ^2^Bioinformatics and Systems Biology, Justus-Liebig-University Giessen, Germany; ^3^Department of Entomology, Michigan State University East Lansing, MI, United States

**Keywords:** *Serratia*, antimicrobial, symbionts, commensal, virulence factors, comparative genomics

## Abstract

Strains of *Serratia marcescens*, originally isolated from the gut lumen of adult female *Anopheles stephensi* mosquitoes, established persistent infection at high rates in adult *A. stephensi* whether fed to larvae or in the sugar meal to adults. By contrast, the congener *S. fonticola* originating from *Aedes triseriatus* had lower infection in *A. stephensi*, suggesting co-adaptation of *Serratia* strains in different species of host mosquitoes. Coinfection at high infection rate in adult *A. stephensi* resulted after feeding *S. marcescens* and *Elizabethkingia anophelis* in the sugar meal, but when fed together to larvae, infection rates with *E. anophelis* were much higher than were *S. marcescens* in adult *A. stephensi*, suggesting a suppression effect of coinfection across life stages. A primary isolate of *S. marcescens* was resistant to all tested antibiotics, showed high survival in the mosquito gut, and produced alpha-hemolysins which contributed to lysis of erythrocytes ingested with the blood meal. Genomes of two primary isolates from *A. stephensi*, designated *S. marcescens* ano1 and ano2, were sequenced and compared to other *Serratia* symbionts associated with insects, nematodes and plants. *Serratia marcescens* ano1 and ano2 had predicted virulence factors possibly involved in attacking parasites and/or causing opportunistic infection in mosquito hosts. *S. marcescens* ano1 and ano2 possessed multiple mechanisms for antagonism against other microorganisms, including production of bacteriocins and multi-antibiotic resistance determinants. These genes contributing to potential anti-malaria activity including serralysins, hemolysins and chitinases are only found in some *Serratia* species. It is interesting that genome sequences in *S. marcescens* ano1 and ano2 are distinctly different from those in *Serratia* sp. Ag1 and Ag2 which were isolated from *Anopheles gambiae*. Compared to *Serratia* sp. Ag1 and Ag2, *S. marcescens* ano1 and ano2 have more rRNAs and many important genes involved in commensal and anti-parasite traits.

## Introduction

Bacteria of the genus *Serratia* are Gram-negative, rod-shaped, facultative anaerobes (Grimont and Grimont, 2006). These bacteria are ubiquitously distributed in soil, sediments, water, plant roots, on surfaces of animals, as well as in the gastrointestinal tract of animals. *Serratia marcescens* or *S. marcescens*-like bacteria have been reported living symbiotically with, and sometimes causing disease in, economically important insects and nematodes (Benoit et al., 1990; Stanley et al., 2012; Wang et al., 2014; Aggarwal et al., 2015; Pei et al., 2015; Chen et al., 2016). For example, *S. marcescens* infection caused acute mortality in mosquitoes and reduced fitness in survivors (Bahia et al., 2014). Some *S. marcescens* strains were pathogenic to apple maggot flies *Rhagoletis pomonella* and to house flies *Musca domestica* (Benoit et al., 1990; Lauzon et al., 2003). Chitinase-producing *S. marcescens* showed insecticidal activity in caterpillars of the moth, *Spodoptera litura* (Aggarwal et al., 2015). Nematodes in soil tolerate infection with *S. marcescens*, and transfer the infection upon encounter to their insect hosts, whence pathology in the insects becomes apparent (Abebe et al., 2011). Pathology in insects infected by *S. marcescens* appears to be related to establishment of disseminated infection in the hemolymph, whilst infection in the gut lumen is tolerated (Nehme et al., 2007).

The physiological activity of *S. marcescens* in the mosquito gut lumen remains largely unknown. However, presence of *S. marcescens* in that lumen is associated with anti-malaria parasite activity, presenting the possibility that manipulation of natural populations of vector mosquitoes with *S. marcescens* infection could control malaria transmission. For example, introduction of a low dose of *S. marcescens* (10^3^ cells/μl) *per os* suppressed *Plasmodium* development in the *Anopheles gambiae* gut (Bahia et al., 2014). In the same study, *in vitro* tests demonstrated that live *S. marcescens* cells or cell-free culture broth inhibited *Plasmodium* ookinete development, a stage that forms in the mosquito gut lumen (Bahia et al., 2014). Moreover, in *Anopheles albimanus*, only 1% of female mosquitoes became infected with *Plasmodium vivax* when *S. marcescens* was simultaneously introduced *per os* in the blood meal, whereas 71% of mosquitoes became infected with advanced stages of the parasite when *S. marcescens* was excluded from the blood meal (Gonzalez-Ceron et al., 2003). The inhibition of *Plasmodium* development in the *Anopheles* midgut by *S. marcescens* could be mediated by multiple mechanisms, such as production of metabolites with anti-parasite properties (Lazaro et al., 2002; Azambuja et al., 2005; Bahia et al., 2014). For example, prodigiosin or its tripyrrole pigment derivatives, produced by various bacteria including *S. marcescens*, demonstrated strong inhibitory activity against *P. falciparum* (Lazaro et al., 2002; Azambuja et al., 2005). Experiments provided evidence that direct contact between *S. marcescens* and *Plasmodium* cells inhibited parasite development in the midgut lumen, an effect possibly mediated by increased expression of a flagellum-specific biosynthesis pathway (Bando et al., 2013). *Serratia marcescens* modulated the mosquito immune system to interfere with the development of malaria parasites (Stathopoulos et al., 2014). Further, genetic variation in immune-related genes influenced the course of infection of *S. marcescens* in *A. gambiae*, involving the peptidoglycan recognition factor, type 3 fibronectin binding proteins, and antibacterial activity of a gustatory Gr9 receptor (Stathopoulos et al., 2014).

Characterizations of the adult *Anopheles* microbiome showed that *Serratia* bacteria were a predominant member of the microbiota in the midgut lumen (Wang et al., 2011; Chen et al., 2016). Genomes of *Serratia* sp. Ag1 and Ag2 isolated from the African malaria vector mosquito *A. gambiae* were recently sequenced and annotated, because of the potential importance of these bacteria to the mosquitoes' immune response, to malaria parasite development, and to pathogenicity of the bacteria to mosquitoes (Bahia et al., 2014; Pei et al., 2015). Nevertheless, comparative genomic and functional analyses are lacking in those *Serratia* forming symbiotic and pathogenic relationships with insect hosts; nor are there detailed studies on interactions between commensal *Serratia* and other bacterial symbionts in mosquito hosts. Given that *S. marcescens* has traits suitable for control of mosquito-borne parasites, a physiological and comparative genome analysis for *S. marcescens* would contribute to (1) development of an efficient paratransgenesis biocontrol strategy (i.e., malaria vector control), (2) incorporation of the effectors/virulence factors from *Serratia* into mosquito symbionts for efficient paratransgenesis systems, and (3) elucidation of the ecophysiology of the diversity of commensals in the mosquito midgut (Bando et al., 2013; Bahia et al., 2014; Aggarwal et al., 2015). Here, we investigated the interaction, persistence and competition amongst different *Serratia* species and strains associated with mosquito gut infection, and with the bacterium *Elizabethkingia anophelis*, another commensal found in that same environment, often together with *S. marcescens*. Further, we tested antimicrobial resistance and hemolytic capabilities to better understand the physiological role of *S. marcescens* as commensals in midgut. Lastly, with the completion of genome sequencing, assembly and annotation, we explored gene repertoire and gene diversity in comparison with other *Serratia* strains and species.

## Materials and methods

### Culture conditions

*Serratia marcescens* strains, designated here ano1 and ano2, were isolated from two female *A. stephensi* (LBT and LB1) in our laboratory colony by aseptic dissection of the midgut using sterile tuberculin syringes and needles, followed by plating midgut contents plus sterile saline on Luria-Bertani agar (BD, USA) (Chen et al., 2016). *Serratia fonticola* strain MSU001 was isolated similarly from a female *Aedes triseriatus* Say from our laboratory colony. History of mosquito strains is reported elsewhere (Chen et al., 2014, 2015, 2016). *Serratia marcescens* strain ano1, *S. marcescens* strain ano2, *Serratia fonticola* strain MSU001 and their derivatives (see below) were cultured in LB by shaking at 28°C. A dual luciferase expression system was used to mark bacterial strains for quantification. A NanoLuc luciferase-tagged *E. anophelis* (SCH814) used in this study was previously established and grown on CYE medium (Chen et al., 2015); the firefly system was developed for strains isolated here (see below). *Escherichia coli* DH5α and *E. coli* λ *pir* were used for cloning and conjugative transfer of DNA, respectively. *E. coli* cells for experiments were grown aerobically in LB broth at 37°C. Bacto agar (Difco, Detroit, MI) was added to a final concentration of 20 g/l. Kanamycin (100 μg/ml) was added for plasmid selection in *E. coli*, but a higher concentration of kanamycin (600 μg/ml) was required for plasmid selection in *Serratia* spp.

### Molecular manipulation

Isolation and purification of bacterial genomic DNA were performed with the Wizard Genomic DNA Purification Kit (Promega, CA, USA). Integrity and quantity of DNA were assessed using gel electrophoresis, a NanoDrop 2000 UV-Vis spectrophotometer (Thermo Scientific, MA, USA) and Qubit 2.0 fluorometer (Life Technologies, MA, USA), respectively.

The firefly reporter vector was developed based on the wide-range plasmid pBBR1 MCS2 (Chen and Hickey, 2011). The *fluc* gene was cloned from vector GL4.50 (Promega, WI) with forward primer Walker219 (aggatcctttaagaaggagatatacatatggaagacgccaaaaacataaag) and reverse primer Walker208 (agcatgcttacaatttggactttccgcccttc) with BamHI and SphI on its 5′ and 3′-end, respectively. The amplicon was gel purified and ligated into the T-easy vector (pSCH955). The insert was released from pSCH955 by the restriction enzymes BamHI and SphI and cloned into the same sites on pBBR1 MCS2 downstream of the P*lac* promoter (pSCH956). Plasmid pSCH956 was introduced into *E. coli S17* lamda *pir* to facilitate conjugation, creating the donor strain for the *fluc* reporter plasmid (SCH981). SCH981 was mixed with recipient *S. marcescens* ano1 or *S. fonticola* MSU001 conjugatively to transfer plasmid pSCH956, leading to firefly reporter strains SCH983 (*S. marcescens*) or SCH985 (*S. fonticola*), respectively.

### Bacterial interactions and luciferase activity determination

Laboratory rearing procedures for *A. stephensi* mosquitoes were previously described (Chen et al., 2015). To investigate persistence of infection of bacteria in host mosquitoes, we performed experiments with *S. marcescens* alone, *S. marcescens* and *E. anophelis* together, or *S. fonticola* and *E. anophelis* together, using strains with NanoLuc and firefly reporters as described above to quantify presence of bacteria in the mosquito gut lumen. The first experiment was designed to investigate bacterial persistence in live larvae, across molts and metamorphosis past the pupal stage into the adult stage, when bacteria were fed to larvae. When larval *A. stephensi* reached 3rd instar, individual reporter bacteria SCH983 (*S. marcescens* with firefly reporter), SCH985 (*S. fonticola* with firefly reporter) or SCH814 (*E. anophelis* with NanoLuc reporter) or combinations of them (SCH814/SCH983 or SCH814/SCH985) were added to sterile water in plastic dishes (final concentration adjusted to approximately 4 × 10^8^ CFU/ml) containing 50 larvae, and larvae allowed to feed on the suspension for 24 h, after which the regular larval food regime (Tetra fish food) was continued. Upon metamorphosis, pupae were retrieved, rinsed with sterile water, and transferred into distilled water in cages for adult emergence. After holding the adults for 4 days (during which time they were provided 10% sucrose solution prepared with sterile water), at least 24 adults were randomly sampled and proceeded as described previously (Chen et al., 2015). A second experiment was conducted to investigate persistence of bacterial infection when introduced to adult mosquitoes. The same bacterial preparations as those described in the larval experiment above were fed to adults (1 day of post-emergence) in 10% sucrose solution. After *ad lib*. feeding for 24 h, the bacterial solution was replaced with fresh, sterile 10% sucrose solution and mosquitoes were sampled at intervals thereafter, and surface-sterilized by immersion in 70% ethanol followed by extensive washings in sterile Milli-Q water. Mosquitoes were homogenized in sterile phosphate buffered saline (PBS), diluted if necessary, and immediately subjected to the Nano-Glo Dual-Luciferase Reporter Assay (Promega, WI).

### Antibiotic susceptibility test

Susceptibility to different antibiotics was tested by minimal inhibition concentration methods in LB broth (European Committee for Antimicrobial Susceptibility Testing of the European Society of Clinical Microbiology and Infectious Diseases, 2003). Cultures were incubated at 28°C for 24 h under aerobic conditions and the OD_600nm_ interpreted according to the manufacturer's instructions. Assays were performed in triplicates.

### Urase production

Urease production was tested by using BBL urase test broth (BD, MD, USA). *Serratia marcescens* strain ano1 or *E. coli* DH5α was inoculated to the broth and incubated for 48 h.

### Hemolytic assays and erythrocyte digestion test

Hemolysin production in *S. marcescens* strain ano1 was tested by inoculating bacterial cells on Remel Blood Agar plate (Thermo Scientific, KS). Hemolytic activity was evaluated following incubation at 28°C for 48 h. *Staphylococcus aureus* and *Elizabethkingia meningoseptica* were used as the positive controls of beta- and alpha-hemolysin, respectively. Bovine whole blood cells (Hemostat Laboratories, CA) were washed with phosphate buffered saline (PBS) and re-suspended in 1 mL of PBS. *Serratia marcescens* strain ano1 (final concentration, 4.5 × 10^8^ cells/mL) was incubated with above washed blood cells for 48 h. The erythrocytes were counted using a hemocytometer under microscopy. The negative control was the same as the treatment without introduction of bacteria.

### Genome sequencing, assembly, and annotation

Next generation sequencing (NGS) libraries were prepared using the Illumina TruSeq Nano DNA Library Preparation Kit following standard procedures recommended by the manufacturer. Completed libraries were evaluated using a combination of Qubit dsDNA HS, Caliper LabChipGX HS DNA and Kapa Illumina Library Quantification qPCR assays. Libraries were combined in a single pool for multiplexed sequencing and this pool was loaded on one standard MiSeq flow cell (v2) and sequencing was performed in a 2 × 250 bp paired end format using a v2, 500 cycle reagent cartridge. Base calling was done by Illumina Real Time Analysis (RTA) v1.18.54 and output of RTA was demultiplexed and converted to FastQ format with Illumina Bcl2fastq v1.8.4.

*De novo* assembly was performed using SPAdes (version 3.9.0) (Bankevich et al., 2012). The reads were assembled using SPAdes (version 3.9.0) and further edited by using DNASTAR (v1.12) (Nurk et al., 2013). Initial prediction and annotation of open reading frames (ORF) and tRNA/rRNA gene prediction were carried out with the Rapid Annotation using Subsystem Technology server (RAST) (Overbeek et al., 2014). Gene annotation was carried out by NCBI Prokaryotic Genome Automatic Annotation Pipeline (PGAAP 3.3) (http://www.ncbi.nlm.nih.gov/genome/annotation_prok/).

### Bioinformatics

The functional categorization and classification for predicted ORFs were performed by RAST server-based SEED viewer (Overbeek et al., 2014). Multi-drug resistance genes were predicted in the Comprehensive Antibiotic Resistance Database (McArthur et al., 2013). Prophage prediction was done with PHAST (Zhou et al., 2011) and Clustered Regularly Interspaced Short Palindromic Repeats (CRISPR) were predicted by CRISPRfinder (Grissa et al., 2007). For genome similarity assessment, average nucleotide identity (ANI) was computed using EDGAR 2.0 (Blom et al., 2016).

The pan genome, core genome, and specific genes of *S. marcescens* ano1 and ano2 were analyzed by comparing with those in 11 representative *Serratia* genomes using EDGAR 2.0 (Blom et al., 2016). The development of pan genome and core genome sizes was approximated using the core/pan development feature. The core genome calculated by EDGAR 2.0 was used to infer a phylogeny for the 13 *Serratia* genomes in this study. The 27,144 amino acid sequences (2,088 per genome) of the core genome were aligned set-wise using MUSCLE v3.8.31 (Edgar, 2004), resulting in a large multiple alignment with 9,015,188 amino acid residues in total (693,476 per genome). This large alignment was used to construct a phylogenetic tree using the neighbor-joining method as implemented in the PHYLIP package (Felsenstein, 1989).

### Accession of the genome sequences

The data from these Whole Genome Shotgun projects have been deposited at DDBJ/ENA/GenBank under the accession. The version described in this paper is version MJVB00000000 and MJVC00000000 for *S. marcescens* ano1 and ano2, respectively. The BioProject designations for this project are PPRJNA340333 and PRJNA340334, and BioSample accession numbers are SAMN05712591 and SAMN05712592 for *S. marcescens* ano1 and ano2, respectively.

### Statistical analyses

Statistical analyses were performed using SAS (version 9.2; SAS Institute, Cary, NC).

## Results

### Colonization and interaction by *Serratia* and *E. anophelis* in mosquitoes

When *S. marcescens* ano1 was fed to 3rd instar *A. stephensi* larvae, the infection persisted to the adult stage for 62.5% (15/24) of adults at 4 days after adult emergence (Table 1), as evidenced by positive luciferase assay relative to negative controls. When *S. marcescens* ano1 was fed in a sugar meal to adult mosquitoes for 24 h, infection rates of the guts were 98.5% (39/40) when dissected 4 days later (Table 1). By contrast, 17.1% (6/35) and 32.5% (13/40) of the guts of *A. stephensi* adults were positive for *S. fonticola* MSU001 when fed to larval and adult stages, respectively, demonstrating that *S. fonticola* isolated from *A. triseriatus* had relatively poorer colonization and persistence than did *S. marcescens* in *A. stephensi*. When *E. anophelis* strain was fed to larvae and adults respectively, 87.5% (35/40) and 100% (40/40) infection rates were observed (Table 1). When *S. marcescens* was introduced together with *E. anophelis* to the adult host by sugar meal at a cell concentration ratio of 1:1, both of the bacteria had similar infection rates (92.5 and 95%, respectively), using the Dual Luciferase assay. These infection rates were comparable to those in adults introduced with a single bacterial species (Table 1). However, when they were co-introduced in the larval stage, the *S. marcescens* infection in *A. stephensi* (22.9%) was lower than that of the single *S. marcescens* species (62.5%), while NanoLuc-labeled *E. anophelis* after feeding to larvae reached an infection rate of 100% in adults whether fed with *S. marcescens* or fed alone. A similar trend was also observed in the co-infection experiment between *S. fonticola* and *E. anopheles* (Table 1). These results further demonstrated that infection rate of *S. fonticola* in *A. stephensi* was lower than that of *S. marcescens* when they were co-introduced at either larval or adult stages, suggesting that *S. marcescens* was co-adapted for infection in *A. stephensi*. Overall, the rank of infection rate to *A. stephensi* for the three selected bacteria was *E. anophelis* > *S. marcescens* > *S. fonticola*.

**Table 1:** Interaction among *S. marcescens* ano1, *S. fonticola*, and *E. anophelis* in larval and adult mosquitoes.

	**Single**	**Single**	**Single**	**Double**		**Double**	
	***E. anophelis***	***S. marcescens***	***S. fonticola***	***S. fonticola***	***E. anophelis***	***S. marcescens***	***E. anophelis***
Larvae 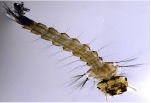	87.5% (35/40)	62.5% (15/24)	17.1% (6/35)	7.5% (3/40)	100% (40/40)	22.9% (8/35)	100% (35/35)
Adults 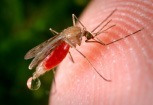	100% (40/40)	98.5% (39/40)	32.5% (13/40)	25% (10/40)	90% (36/40)	92.5% (37/40)	95% (38/40)

*Individual S. marcescens ano1 (Firefly reporter), S. fonticola (Firefly reporter), and E. anophelis (NanoLuc reporter) or combinations of them were orally fed to larval or adult mosquitoes. The bacterial infection in mosquito was quantified by using Dual-Luciferase Reporter Assay kit as described in Materials and Methods. The infection rates are shown as percentages on the top and the numbers in brackets are individuals analyzed*.

### Genome features and phylogenetic inferences

The assembly of strain ano1 contained 44 contigs with a size of 5.45 Mbp (Table 2). The assembly of strain ano2 contained 71 contigs with a size of 5.47 Mbp (Table 2). The ano1 genome included 5,082 coding sequences (CDS) and 119 RNA genes. The ano2 genome included 5,058 CDS and 118 RNA genes (Table 2). Among the 13 *Serratia* genomes selected for comparative analysis here, ano1 or ano2 had the most CDSs (Table 2). The average GC content for ano1 and ano2 was 59.6 and 59.5%, respectively, consistent with other *S. marcescens*; however, the average GC content in *S. marcescens* was much higher than that in other *Serratia* spp. (Table 2). No plasmid sequence was found in either ano1 or ano2, congruent with our inability to isolate plasmids from the two *S. marcescens* strains. RAST analysis showed that ano1 and ano2 have at least 589 and 588 subsystems, respectively (Figure S1).

**Table 2:** Genome features for selected *Serratia* spp. or *Serratia*-like bacteria.

**Strains**	**Genome size (Mb)**	**Gene count**	**CRISPR count**	**GC (%)**	**Predicted CDS**	**Total RNA**	**Accession number**	**Isolation site**	**References**
*S. marcescens* ano1	5.45	5,131	0	60	5,082	119	MJVB00000000	Mosquito	This study
*S. marcescens* ano2	5.47	5,176	0	60	5,058	118	MJVC00000000	Mosquito	This study
*Serratia* sp. Ag1	5.35	5,061	5	52	4,773	85	JQEI00000000	Mosquito	Pei et al., 2015
*Serratia* sp. Ag2	5.32	5,031	5	52	4,706	85	JQEJ00000000	Mosquito	Pei et al., 2015
*S. marcescens* Db11	5.11	4,831	0	60	4,721	110	HG326223.1	Drosophila	Iguchi et al., 2014
*S. marcescens* MCB	5.30	5,048	0	59	4,874	174	JPQY00000000	Nematodes	Serepa and Gray, 2014
*Serratia* sp. TEL	5.00	4,732	2	59	4,544	188	KP711410	Nematodes	Lephoto and Gray, 2015
*S. marcescens* FGI94	4.86	4,609	4	59	4,438	171	CP003942	Fungus garden	Li P. et al., 2015
*S. plymuthica* S13	5.47	5,125	0	56	5,018	107	NC_021659	Plant	Müller et al., 2013
*S. proteamaculans* 568	5.50	5,063	0	55	4,954	109	CP000826	Plant	Abebe-Akele et al., 2015
*S. liquefaciens* ATCC 27592	5.28	5,023	0	55	4,914	109	CP006252	Milk	Nicholson et al., 2013
*S. marcescens* WW4	5.24	4,919	0	60	4,818	101	CP003959	Paper machine	Chung et al., 2013

**Figure 1:**
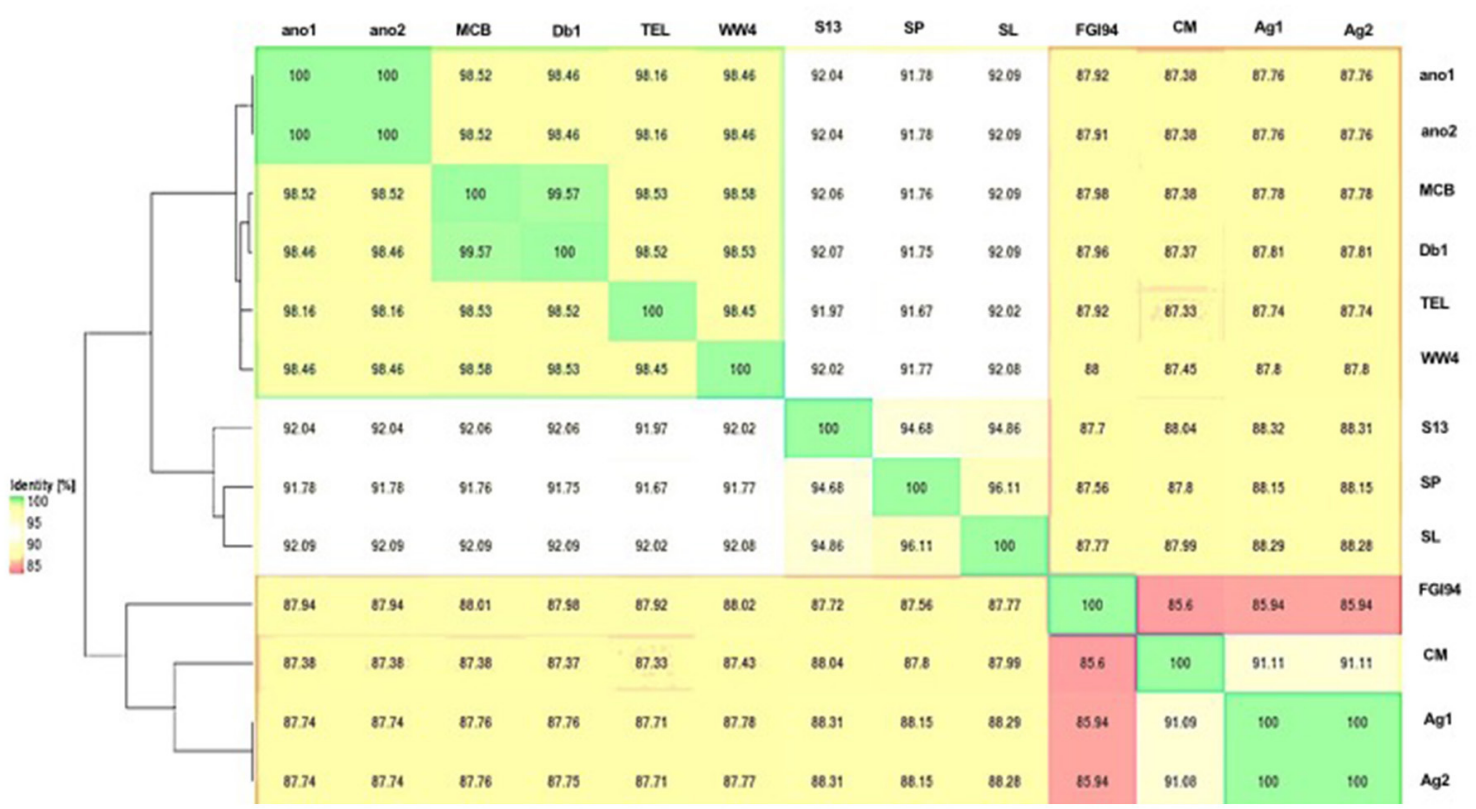
Average nucleotide identity dendogram for the selected *Serratia* spp. ANI matrix generated by complete genome sequences was used to calculate an ANI divergence dendrogram. The hosts for these isolates were shown on the right panel. Ano1, *S. marcescens* ano1; Ano2, *S. marcescens* ano2; Ag1, *Serratia* sp. Ag1; Ag2, *Serratia* sp. Ag2; MCB, *S. marcescens* MCB; Db11, *S. marcescens* Db11; TEL, *Serratia* sp. TEL; FGI94, *S. marcescens* FGI94; Sp, *S. plymuthica* S13; Sl, *S. liquefaciens* ATCC 27592; WW4, *S. marcescens* WW4.

The difference in genome features between ano1 and ano2 was not remarkable (Table 2, Figure 1, and Figure S2). The calculated ANI between ano1 and ano2 shows 100% identity, indicating that *S. marcescens* ano1 and ano2 were the same strain (Figure 1). ANI values indicated that these two isolates belong to *S. marcescens* species as they were more than 95% identical to those in *S. marcescens* MCB (Serepa and Gray, 2014) and *S. marcescens* Db1 (Flyg et al., 1980), previously isolated from nematodes and fruit flies, respectively (Figure 1). *Serratia* sp. TEL could be assigned to *S. marcescens* because it had a high ANI value (>95%) with those of *S. marcescens* MCB and *S. marcescens* Db1. It is interesting that, compared to that in *S. marcescens* FGI94, low ANI values (<89%) in most of the selected *Serratia* were found, highlighting that different *Serratia* sp. exist in various insects. Phylogenetic trees (based on genome analysis) showed that ano1 and ano2 were most closely related to *Serratia* sp. TEL and *S. marcescens* WW4 (Figure S3). It is interesting that *Serratia* sp. Ag1 and Ag2 have more CRISPR elements than do other *Serratia* (Table 2). We did not detect any CRISPR elements in ano1 and ano2. Remarkably, *Serratia* sp. Ag1 and Ag2 isolated from mosquito *A. gambiae* have different genome features compared to ano1 and ano2, such as GC content, ANI values and predicted CDS (Table 2, Figure 1, and Figure S3 and see below).

*Serratia marcescens* strains ano1 and ano2 had 5 predicted prophages (Table S1). Two of the prophages (prophage 3 and 4) were possibly complete because they consisted of tails, heads, portals, integrases, lysins and other component proteins involving phage structure and assembly. Mosquito isolates Ag1 and Ag2 have two incomplete prophages. The number of predicted prophages in other *Serratia* ranged from 1 to 8. Collectively, these prophages varied in size and gene organization, indicating their diversity in *Serratia* (Table S1).

### Gene repertoire of *S. marcescens*

**Figure 2:**
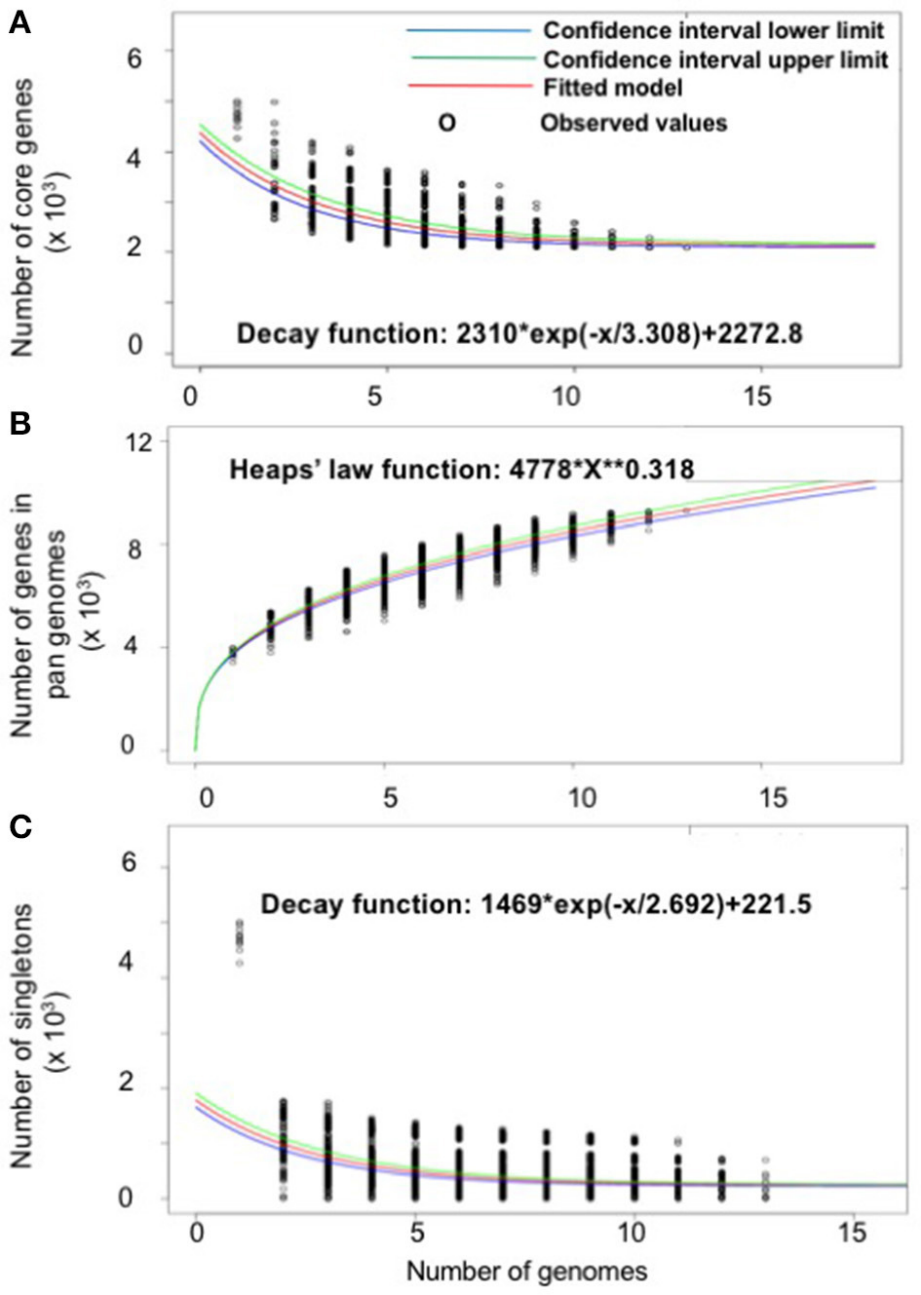
Pan, core, and singleton genome evolution according to the number of selected *Serratia* genomes. **(A)** Number of genes (pan-genome) for a given number of genomes sequentially added. **(B)** Number of shared genes (core genome) as a function of the number of genomes sequentially added. **(C)** Number of unique genes (accessory genome) for a given number of genomes sequentially added.

The gene repertoire of the selected *Serratia* species was analyzed using their ubiquitous genes (core genome) and different homologous genes families (pan-genome) amongst the selected *Serratia* genomes. The pan-genome plot shows that the power trend line had not reached a plateau (Figure 2A), demonstrating that *Serratia* displays an open pan-genome. *Serratia* core genome analysis showed that the number of shared genes decreased with the addition of the input genomes and was predicted to converge against 2,127 (see Figure 2B). Singleton development plot data indicated that up to 240 new genes could be expected with every newly sequenced genome (Figure 2C). The core genome for the 13 selected *Serratia* was calculated to be 2,088 CDS per genome; given the assumption that the approximation slightly over-predicts the real core genome size, the current core genome likely represents the *Serratia* genus quite well.

**Figure 3:**
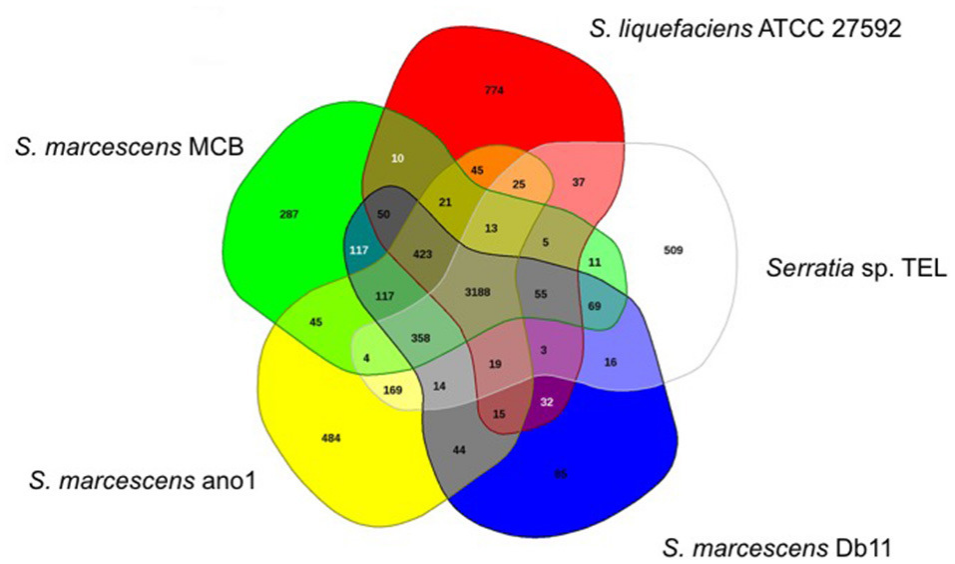
Venn diagram of shared and unique genes in the selected *Serratia*. The unique and shared genome among the compared genomes was determined by a dual cutoff of 30% or greater amino acid identity and sequence length coverage of at least 70%. Analysis was done using the MAUVE genome alignment tool. 1, *S. liquefaciens*; 2, *S. marcescens* MCB; 3, *S. marcescens* ano1; 4, *S. marcescens* Db11, and 5, *Serratia* sp. TEL.

*Serratia marcescens* ano1 and ano2 shared at least 4985 genes (data not shown). Only 13 and 12 genes were uniquely present in strains ano1 and ano2, respectively, a result within the error rate of incomplete sequencing and likely artifact. Also, among its relatives, the ano1 genome shared in common genes ranging from 2936 to 3749 in number, with the lowest number shared with *Serratia* sp. Ag2, indicating diverse physiological functions in the selected *Serratia* (Figure 3 and Figure S4). For example, *S. marcescens* ano1 shared at least 4198, 4178, 4172, and 3788 common genes with strains *S. liquefaciens, S. marcescens* Db11, *S. marcescens* MCB and *Serratia* sp. TEL, which accounts for approximately 84.0, 83.6, 83.5, and 75.8% of its encoding genes, respectively; the above 5 selected *S. marcescens* shared 3188 common genes (Figure 3).

### Antimicrobial resistance

*Serratia marcescens* ano1 was resistant to nearly all of the selected antibiotics including class β-lactams, aminoglycosides, tetracycline, aminoglycosides, macrolides, glycopeptides, and ansamycins, showing that ano1 is a multi-drug resistant strain (Table 3). At least 18 predicted enzymes/proteins conferring antibiotic resistance were predicted by CARD and RAST SEED subsystem (Figure S1 and Table S2). The predicted proteins include those conferring antibiotic resistance to β-lactams, fosfomycin, mupirocin, polymyxin, aminocoumarin, aminoglycoside, isoniazid and fluoroquinolone. Furthermore, up to 37 efflux pumps were identified, possibly contributing to tetracycline, rifampin, aminocoumarin, aminoglycoside, fluoroquinolone, macrolide as well as β-lactam resistance (Table S2). Comparative study of *Serratia* genomes showed that all of them have potential resistance against four classes of antibiotics: fluoroquinolone, polymyxin, mupirocin, and fosfomycin.

**Table 3:** Antibiotic susceptibility tests in *S. marcescens*.

**Drug class**	**Antibiotics**	Susceptibility^*^
Ansamycins	Rifampicin	R
Glycopeptides	Vancomycin	R
Macrolides	Erythromycin	R
	Chloramphenicol	R
	Pimaricin	R
Aminoglycosides	Streptomycin	R
	Phenylmethanesulfonyl fluroide	R
	Phosphomycin	R
Tetracyclines	Tetracycline	R
β-lactam	Ampicillin	R
	Carbenicillin	R
	Cephalosporin	R
Aminoglycosides	Kanamycin	R

* *R refers to resistant. The final concentration for tested antibiotics is 10 μg/ml*.

Most of the *S. marcescens* strains had genes encoding antimicrobial compound synthesis proteins such as hydrogen cyanide (OHT38223), bacteriocin (colicin V, OHT33745) and pyoverdine (OHT34953) (also see next), indicating that they have potential to inhibit the growth of similar or closely related bacterial strain(s) (Table S3). Ano1 and ano2 carried the gene cluster of biosynthesis of bacteriocin, consisting of 19 opening reading frames (ORFs). Most of them were conserved among the *Serratia* genomes examined here, including those isolated from mosquitoes and nematodes (Table S3). It is noteworthy that prodigiosin gene synthesis clusters were absent in *S. marcescens* ano1, also lacking in several insect symbionts (Table S4).

### Virulence factors predicted in *Serratia*

Strains ano1 and ano2 had a probability of acting as a pathogen of 72.9% according to the probability values assigned by PathogenFinder (Cosentino et al., 2013). Ano1 genome matched 41 pathogenic families and 12 non-pathogenic families in the database (data not shown). Furthermore, a total of 37 putative virulence factors were predicted by VFDB, which mainly account for flagella formation, lipooligosaccharide (LOS), attacking enzymes, iron uptake and transportation, different secretion systems and hemolysis of red blood cells (Table 4). It is interesting that several predicted virulence factors in ano1 and ano2 such as *gluP, manC, urase, shu*, and *fleQ* were either missed or showed low identity (<40%) in mosquito symbionts *Serratia* sp. Ag1 and Ag2 (Table 4). For example, *gluP* (encoding L-fucose:H+ symporter permease, BGV45_11240), participates in chronic infection and acts an important virulence factor in *Brucella abortus* (Xavier Mariana et al., 2013). *manC*, a mannose-1-phosphate guanylyltransferase gene (forming O-antigen) (Lee et al., 1992), was absent in Ag2 though there is a *manC-*like gene in Ag1 showing a significantly low identity (37%). This *manC* gene is absent in environmental isolate *S. marcescens* FGI94. An iron transporter gene, *shu* (Mourer et al., 2015), existed in most of the selected *Serratia* strains (>87%) while it was absent in those in Ag1 and Ag2 (Table 4).

**Table 4:** Prediction of virulence factors in *Serratia* spp.

	**Locus ID**	**Gene**	**Predicted gene products**	**Selected** ***Serratia*** spp. and their isolation sites^*^										
				**Mosquitoes**				**Fly**	**Nematodes**		**Fungi**	**Plants**	**Milk**	**Paper**
				**Ano1**	**Ano2**	**Ag1**	**Ag2**	**MCB**	**Db11**	**TEL**	**FGI94**	**Sp**	**Sl**	**WW4**
**FLAGELLA FORMATION**														
	OHT36767	*flgN*	Flagella synthesis	100	100	63	63	98	98	99	67	83	82	98
	OHT36770	*flgB*	Basal-body rod protein	100	100	82	82	98	98	97	87	89	90	98
	OHT36762	*flhB*	Biosynthetic protein	100	100	82	82	99	99	99	87	0	93	99
	OHT36750	*motA*	Motor protein	100	100	95	95	99	100	99	96	96	98	100
	OHT38566	*fleQ*	Transcriptional regulator	100	100	48	48	94	94	48	47	47	49	94
	OHT36783	*fliQ*	Biosynthetic protein	100	100	98	98	100	100	100	94	94	95	100
	OHT36774	*flgF*	Basal-body rod protein	100	100	86	86	99	99	97	88	95	94	97
	OHT36782	*fliR*	Biosynthetic protein	100	100	84	84	99	99	99	87	93	92	99
	OHT42321	*tsr*	Chemotaxis protein I	100	100	56	56	99	99	98	88	92	92	98
**EXTRACELLULAR CELL STRUCTURES**														
LPS	OHT35016	*gluP*	L-fucose:H+ symporter permease	100	100	0	0	99	99	98	86	91	91	99
	OHT39654	*fabZ*	(3R)-hydroxymyristoyl ACP dehydratase	100	100	98	98	100	99	100	98	97	97	99
LOS	OHT38306	*msbA*	Lipid transporter ATP-binding/permease	100	100	93	93	99	99	99	94	93	94	99
	OHT32281	*msbB*	lipid A biosynthesis	100	100	93	93	100	100	29	94	93	94	100
	OHT38430	*galE*	UDP-glucose 4-epimerase	100	100	79	79	77	94	92	74	87	88	94
	OHT38979	*manC*	O-antigen synthesis	100	100	37	0	59	61	60	0	60	59	61
	OHT39625	*lpxD*	UDP-3-O-(3-hydroxymyristoyl) glucosamine N-acyltransferase	100	100	96	96	99	99	99	95	96	97	99
	OHT41381	*rfaF*	ADP-heptose-LPS heptosyltransferase II	100	100	90	90	98	98	98	89	91	90	99
Fimbriae	OHT32112	*lpfB*	Long polar fimbrial chaperone protein LpfB	100	100	75	75	38	56	99	66	86	42	53
	OHT35997	*papC*	Usher protein PapC	100	100	60	60	75	75	75	73	74	65	81
**IRON METABOLISM**														
	OHT36555	*entA*	2,3-dihydro-2,3-dihydroxybenzoate dehydrogenase	100	100	68	68	99	99	100	69	66	67	99
	OHT33653	*fepG*	Iron-enterobactin ABC transporter permease	100	100	64	64	99	99	98	63	63	66	98
	OHT37918	*fes*	Ferric enterobactin esterase	100	100	72	72	98	98	95	78	69	69	99
	OHT35700	*shuU*	ABC transport system	100	100	39	39	99	99	99	85	95	93	99
	OHT38589	*fbpC*	Iron(III) ABC transporter	100	100	87	87	98	98	52	88	93	91	99
**SECRETION SYSTEM**														
	OHT38930	*xcpR*	T2SS protein E	100	100	64	64	48	48	94	66	87	47	48
	OHT36978	*hsiJ1*	T6SS protein HsiJ1	100	100	80	80	99	99	99	93	0	0	99
	OHT37058	*mtrD*	T3SS protein MtrD	100	100	91	91	99	99	99	90	93	95	99
**REGULATION**														
	OHT35771	*caf1R*	F1 operon positive regulatory protein	100	100	74	74	98	98	97	77	81	79	97
	OHT34240	*pilR*	Two-component response regulator	100	100	89	89	98	98	95	87	88	89	95
	OHT36991	*chpD*	Transcriptional regulator	100	100	81	81	99	99	98	79	85	81	98
	OHT37846	*fleR/flrC*	σ^54^ response regulator	100	100	96	96	100	100	99	96	96	96	99
**ENZYMES**														
	OHT42068	*ureA*	Urease α subunit	100	100	0	0	0	0	95	0	0	0	0
	OHT35629	*aprA*	alkaline metalloproteinase	100	100	59	59	97	97	96	62	61	82	97
**OTHERS**														
	OHT35425	*IlpA*	Immunogenic lipoprotein A	100	100	69	69	98	98	98	69	91	97	98
	OHT33698	*ccmB*	Cytochrome c biogenesis	100	100	93	93	99	99	99	95	95	94	98
	OHT41640	*algU*	Alginate biosynthesis protein	100	100	100	100	100	100	100	99	100	99	100

* *Ano1, S. marcescens ano1; Ano2, S. marcescens ano2; Ag1, Serratia sp. Ag1; Ag2, Serratia sp. Ag2; MCB, S. marcescens MCB; Db11, S. marcescens Db11; TEL, Serratia sp. TEL; FGI94, S. marcescens FGI94; Sp, S. plymuthica S13; Sl, S. liquefaciens ATCC 27592; WW4, S. marcescens WW4. The protein sequences from S. marcescens ano1 were used to blast (blastP) against those in the other selected Serratia species. The gene product is regarded as absence if the identity is below 60%*.

### Chitinases

At least 4 different chitinase genes encode chitinase A (OHT33328 and OHT32204), chitinase B (OHT36384) and chitinase C (OHT38806) in *S. marcescens* ano1 (Table 5). ChiA digests chitins from the reducing end while ChiB works on the chitin chain from the non-reducing end, indicating that they are processive enzymes (Suzuki et al., 2002; Orikoshi et al., 2005; Vaaje-Kolstad et al., 2013). Instead, ChiC acts as an endo-acting enzyme which cuts chitin chain in the middle (Vaaje-Kolstad et al., 2013). ChiC exists in most of the selected *Serratia*. However, an efficient chitin degradation requires a synergistic action of ChiA, ChiB, and ChiC in *S. marcescens* (Orikoshi et al., 2005; Vaaje-Kolstad et al., 2013). Besides the different catalytic domains (all belonging to GH18 superfamily) (Tian et al., 2014), ChiA1, ChiB and ChiC chitinases have various accessory functional domains (Table 5) such as fibronectin type III (OHT33328), chitin-binding (OHT38806), or cellulose-binding (OHT36384) domains while ChiA2 has only catalytic one (OHT32204) (Tran et al., 2011). Previous studies showed that CBD or chitin-binding domains greatly affected the catalytic activity and substrate affinity in chitinases (Dahiya et al., 2006; Tian et al., 2014). Therefore, the chitinases with various functional domain(s) may provide different physiological roles in *S. marcescens* (Dahiya et al., 2006). The four chitinase genes dispersedly spread in *S. marcescens* ano1 genomes, rather than cluster together to form an operon (data not shown). Such arrangement indicates that they may be induced under the different conditions and regulated by different mechanisms. No genes encoding chitinase (cutoff, 60% identity) were found in *Serratia* sp. Ag1 and Ag2, indicating that they have a poor ability to use chitin as carbon or nitrogen sources from environment or in insects. As expected, in *S. marcescens* FGI94, a lack of ChiA1 and ChiB as production of large amount of chitinase will disrupt its symbiotic relationship with fungi (by degrading chitins in fungal cell walls; Li P. et al., 2015). Instead, similar to ano1 or ano2, Db1, MCB, and TEL have ChiA1, ChiB, and ChiC. WW4 has the complete chitin degradation enzyme system, which is consistent with its living niche (paper machine) where the plant materials are abundant (Chung et al., 2013).

**Table 5:** Comparison of chitinases in selected *Serratia* spp.

**Chitinases**	**Mosquitoes**				**Files**	**Nematodes**		**Fingi**	**Plants**	**Milk**	**Paper**
	**Ano1**	**Ano2**	**Ag1**	**Ag2**	**MCB**	**Db11**	**TEL**	**FGI94**	**Sp**	**SL**	**WW4**
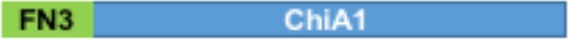	100	100	0	0	99	99	99	0	94	95	99
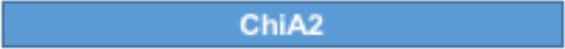	100	100	0	0	96	96	28	0	86	84	99
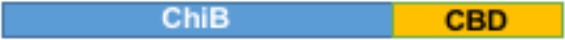	100	100	0	0	99	99	99	0	94	93	99
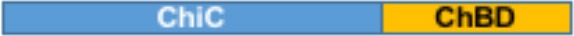	100	100	0	0	98	97	98	83	86	87	99

^*^The left panel shows the organization of the conserved accessory and catalytic domains in various chitinases. The length is not scaled. FN3, fibronectin type III domain. CBD, cellulose-binding domain. ChBD, chitin-binding domain. Ano1, S. marcescens ano1; Ano2, S. marcescens ano2; Ag1, Serratia sp. Ag1; Ag2, Serratia sp. Ag2; MCB, S. marcescens MCB; Db11, S. marcescens Db11; TEL, Serratia sp. TEL; FGI94, S. marcescens FGI94; Sp, S. plymuthica S13; Sl, S. liquefaciens ATCC 27592; WW4, S. marcescens WW4. The protein sequences from S. marcescens ano1 were used to blast (blastP) against those in the other selected Serratia species. The gene product is regarded as absence if the identity is below 60%.

### Hemolysins and serralysins

**Figure 4:**
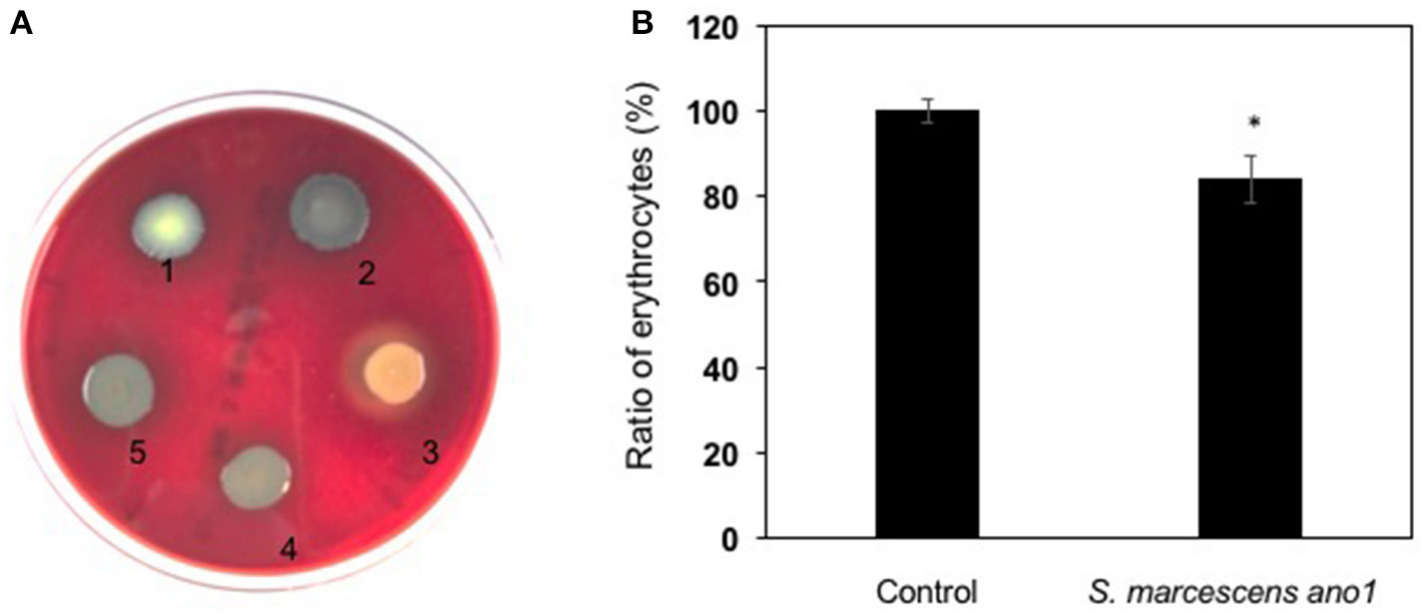
Demonstration of alpha-hemolysin production and lysis of erythrocytes by *S. marcescens*. **(A)** Demonstration of hemolysin production in *S. fonicola* MSU001 (colony1), *S. marcescens* ano1 (colony 2), *S.aureus* (colony 3, beta-hemolysin control), *E. meningoseptica* (colony 4, alpha-hemolysin control), and *S. marcescens* ano1 (colony 5). **(B)** Lysis of the red blood cells by *S. marcescens* ano1. The control was the same as the treatment except no bacterial cells were inoculated. The asterisk indicates there was a significant difference between the control and treatment (student *t*-test, *p* < 0.01). Values are means of single measurements from triplicate cultures (±standard deviations).

*Serratia marcescens* ano1 and ano2 were hemolytic strains with alpha-hemolysin activity (Figure 4A). Red blood cell lysis experiment was further conducted by inoculating *S. marcescens* ano1 in bovine whole cells (Figure 4B). At least 16% of erythrocytes were disrupted within 48 h (Figure 4B). The commensal *S. marcescens* living in mosquito midgut may not utilize the hemolysin(s) for pathogenesis unless they accidently invade the host through liaison and enter the insect hemolymph (Chen et al. unpublished data).

Serralysin genes are present in most of the insect associated *Serratia* (*S. marcescens* MCB, *S. marcescens* Db1 and *Serratia* sp. TEL) with high identity in amino acid sequences (>60%) (Table 6). *S. marcescens* ano1 possesses at least 5 genes encoding serralysins or serralysin-like proteins (OHT33392, OHT35629, OHT35861, OHT36525, and OHT38015) (Table 6). It is interesting that *Serratia* sp. Ag1 and Ag2 have only two of them (Table 6). At least three serralysin genes are found in plant or fungi symbionts *S. marcescens, S. plymuthica* S13, and *S. marcescens* FGI94, respectively. In this study, we identified an additional one based on the genome mining method (Table 6).

**Table 6:** Comparison of serralysins in selected *Serratia* spp.^*^

	**Mosquitoes**				**Flies**	**Nematodes**		**Fungi**	**Plants**		**Paper**
**Serralysin**	**Ano1**	**Ano2**	**Ag1**	**Ag2**	**MCB**	**Db11**	**TEL**	**FGI94**	**Sp**	**Sl**	**WW4**
OHT33392	100	100	81	81	98	98	98	81	90	93	97
OHT38015	100	100	61	61	63	63	63	61	71	74	95
OHT35629	100	100	59	59	97	97	96	62	61	82	97
OHT36525	100	100	53	53	92	95	95	53	53	53	95
OHT35861	100	100	48	48	99	99	98	49	46	78	99

* *Ano1, S. marcescens ano1; Ano2, S. marcescens ano2; Ag1, Serratia sp. Ag1; Ag2, Serratia sp. Ag2; MCB, S. marcescens MCB; Db11, S. marcescens Db11; TEL, Serratia sp. TEL; FGI94, S. marcescens FGI94; Sp, S. plymuthica S13; Sl, S. liquefaciens ATCC 27592; WW4, S. marcescens WW4. The protein sequences from S. marcescens ano1were used to blast (blastP) against those in the other selected Serratia species. The gene product is regarded as absence if the identity is below 60%*.

### Ureases

*ure*A, encoding an α-subunit ureases, was predicted as the virulence factor (Table 3). Among the selected *Serratia*, only ano1, ano2 and TEL carry *ureA* (Table 3). Further examination of the three selected *Serratia* genomes showed that there is an operon consisting of 9 genes possibly involved in urea metabolism (Figure S5). *Serratia marcescens* strain ano1 was confirmed to urease production positive (data not shown), indicating that the “*ure*” operon is functional in ano1. In this “*ure*” operon, three genes (*ure* A, B, and C) were predicted to encode α, β, and γ subunits, respectively (Carlini and Ligabue-Braun, 2016). Genes *ure* D, E, F, and G encode urease accessory proteins that participate in assembly and activation of ureases (Carlini and Ligabue-Braun, 2016). Additionally, urea transporter (*utp*) and nickel transporter (*nix*A) genes immediately locate downstream of urease structure protein or accessory protein encoding genes (Carlini and Ligabue-Braun, 2016). However, the *ure* operon is only found in 14 out of 101 *Serratia* genomes publically available in the IMG/MER (cutoff date, Dec.1, 2016). We found mosquito or nematode-associated *Serratia* (ano1, ano2, TEL) with this operon.

### Iron and heme metabolism

Enterobactin synthesis gene clusters and their organization were conserved in all *S. marcescens* genomes (Table 7). Several operons encoding ferrichrome ABC transporters (*fhu* operon), ferric siderophore ABC transporters (*fep* operon) and ferric citrate outer membrane transporters (*fec* operon) were found (Table 7). *Serratia marcescens* ano1 and ano2 had a delicate set of heme uptake/storage systems (Table 7). The *has* operon encoding the heme uptake system includes an RNA polymerase sigma factor (OHT42022.1), putative iron sensor protein (OHT42023.1), TonB-dependent heme receptor (OHT42024.1), hemophore HasA (OHT42025.1), peptidase (OHT42071.1), hemolysin transporter (OHT42026.1), and TonB-like protein (OHT42027.1). This system ensures that, after heme/hemin is released from hemoglobins or other iron containing proteins, they can be efficiently processed, stored and transported. The ferric uptake regulator (Müller et al., 2013) has a key role in modulating iron uptake, and the genome of *S. marcescens* strain ano1 and ano2 is predicted to encode a Fur protein (OHT38796.1). However, the *fur* gene is not close to any iron metabolism genes in *S. marcescens* (data not shown).

**Table 7:** Iron metabolism genes in selected *Serratia* spp.^*^

**Iron-utilization system**	**Genes**	**Predicted gene products**	**Ano1**	**Ano2**	**Db11**	**FGI94**	**Sp**	**TEL**	**MCB**	**Ag1**	**Ag2**	**Sl**	**WW4**
**The ent operon and entA- and entD-homologs**													
	*entFSCEB*	Enterobactin synthase subunit F, enterobactin exporter, isochorismate synthase,enterobactin synthase subunit E & isochorismatase	100	100	99	84	82	97	99	81	81	83	98
			100	100	99	54	59	99	99	59	59	59	99
	*pswP*	Putative 4'-phosphopantetheinyl transferase (entD homolog)	100	100	94	54	80	94	96	64	64	77	91
**A gene cluster for siderophore synthesis**													
		Predicted transporter, siderophore synthetase CbsF homolog, putative siderophore biosynthesis protein & ferric siderophore esterase	100	100	100	94	57	100	100	60	60	57	99
**The fhu operon**													
	*fhuBDC*	Ferrichrome ABC transporter (permease, substrate-binding & ATP-binding proteins)	100	100	94	72	82	96	96	0	0	84	93
	*fhuA*	Ferrichrome outer membrane transporter	100	100	96	56	73	93	96	0	0	75	94
**The fep operon**													
	*fepBDGC*	Ferric siderophore ABC transporter (substrate-binding, permease, & ATP-binding proteins)	100	100	94	64	61	99	94	54	54	53	96
**The fec operon**													
	*fecARI*	Ferric citrate outer membrane transporter, transmembrane signal transducer & RNA polymerase sigma factor	100	100	91	0	0	0	93	0	0	67	94
**The has operon**													
	*hasIERADEB*	RNA polymerase sigma factor, putative iron sensor protein, TonB-dependent heme receptor, hemophore HasA, heme acquisition ABC transporter (ATPbinding/permease & substrate-binding proteins) & TonB-like protein	100	100	100	0	0	0	100	0	0	0	99
**The hem operon**													
	*hemVUTSR*	Hemin ABC transporter (ATP-binding, permease, & substrate-binding proteins), hemindegrading protein & TonB-dependent hemin receptor	100	100	99	81	81	83	97	55	55	83	100
	*hemP*	Hemin uptake protein	100	100	97	89	87	87	97	0	0	87	100
**The hms operon**													
	*hmsSRFH*	Hemin storage system (HmsS, HmsR, HmsF, & HmsH proteins)	100	100	0	0	0	0	0	0	0	0	100

* *Ano1, S. marcescens ano1; Ano2, S. marcescens ano2; Ag1, Serratia sp. Ag1; Ag2, Serratia sp. Ag2; MCB, S. marcescens MCB; Db11, S. marcescens Db11; TEL, Serratia sp. TEL; FGI94, S. marcescens FGI94; Sp, S. plymuthica S13; Sl, S. liquefaciens ATCC 27592; WW4, S. marcescens WW4. The protein sequences from S. marcescens ano1 were used to blast (blastP) against those in the other selected Serratia species. The gene product is regarded as absence if the identity is below 60%*.

## Discussion

We report here that mosquito-commensal *S. marcescens* bacteria have many genes encoding various virulence factors such as flagella formation, lipooligosaccharide (LOS), iron uptake and transportation, serralysins, hemolysins, and chitinases, indicating that they have great potential to disrupt the development of malaria parasites through different avenues (Lazaro et al., 2002; Gonzalez-Ceron et al., 2003; Azambuja et al., 2005; Bahia et al., 2014). Regardless of the mechanisms, the ability to colonize the midgut and persist over time must precede any anti-parasite activity (Bahia et al., 2014). By using *S. marcescens* ano1 as a representative bacterium to study the interaction between gut bacteria and mosquito, we further demonstrated commensal isolate *S. marcescens* ano1 was able to successfully re-colonize and transstadially persist in the *A. stephensi* mosquito gut. This bacterium can be introduced alone or in combination with other microbes such as *E. anophelis* as a commensal cocktail without substantial inhibition of the members of the consortium, as shown in the bacteria-bacteria interaction experiment (Table 1). Moreover, this isolate was amenable to genetic manipulation (exemplified by expression of reporter genes), opening the possibility to utilize *S. marcescens* for paratransgenic vector control. These properties are extremely important for developing paratransgenic reagents in control of malaria parasite transmission and understanding the bacterial interactions with other gut symbionts as well as the host mosquito.

Biofilm formation is important for establishing microbe-insect symbiosis (Kim et al., 2014). *Serratia marcescens* and *E. anophelis* had better capability to infect and colonize *A. stephensi* than did *S. fonticola* (Table 1). We speculate that adherence and biofilm formation characteristics in *S. marcescens* and *E. anophelis* possibly contribute to their successful colonization in *A. stephensi*. *In vitro* studies showed that biofilm production in *S. marcescens* was two times higher than that in *S. fonticola* (Hamieh et al., 2015). Previous results showed that some specific genes in commensals modulated the biofilm growth and thus facilitated the bacteria-insect symbiosis (Kim et al., 2014; Powell et al., 2016). Biofilms in *S. marcescens* are tightly controlled by a set of sophisticated system including quorum sensing, type 1 fimbriae, and carbon and nitrogen sources (Labbate et al., 2007; Shanks et al., 2007). Shanks et al. (2007) reported that disruption of type I fimbrial genes in *S. marcescens* resulted in severe deficiencies in biofilm formation (Shanks et al., 2007). In the same study, their results showed that biofilm formation was remarkably affected by a mutation of *oxyR*, a transcription factor participating in regulate oxidative stress response (Shanks et al., 2007). Moreover, the bacterial biofilm formation in insect midgut was greatly influenced by gut environment that is frequently changed with the host developmental stages and diet types (Wang et al., 2011; Chen et al., 2016). Our results and others also showed that blood meals dramatically influenced the composition of microbial community, demonstrating that the bacterial ability to respond to the dramatic stress determines their survival in mosquito gut (Wang et al., 2011; Chen et al., 2016). Li et al. reported that the addition of hemoglobin enhanced the attachment of *E. anophelis* to the substratum of the matrix and biofilm biovolume rather than iron (Li Y. et al., 2015). Further, our preliminary data showed that transposon-mediated mutation of a *Bacteriodes* aerotolerance (Bat) protein gene in *E. anophelis* resulted in a low biofilm formation and also inability to colonize *A. stephensi* (Chen et al. unpublished data). However, it remains unclear if there are different gene determinations for biofilm formation in our *A. triseriatus*-isolate *S. fonticola* MSU001 due to the lack of the genome sequences. Future comparative genomic and functional analysis among *S. marcescens ano1, S. fonticola* MSU001, and *E. anophelis* MSU001 will provide better insights into the molecular mechanisms involving in bacterial biofilm formation and colonization.

Bacterial shift from one host to another will encounter the different stressors. To successfully colonize the mosquito host gut, microbes should circumvent: (1) digestion and destructive factors such as secreted lysozyme, antibacterial peptides, and other immune factors from the host; (2) inhibition effects from other microbes (e.g., antibiotics); and (3) stressors such as nutrient limitation and high pH in the mosquito gut. Previous studies showed that gut microbes such as *S. marcescens* and *Elizabethkingia* could tolerate high pH and/or resist digestion (Chen et al., 2015, 2016). Coexistence with predominant bacteria showed that *Serratia* may evolve to cooperate their activity in a niche such as mosquito midgut. Unfortunately, the detailed molecular mechanisms involved in bacterial survival in mosquito hosts are poorly investigated (Dillon and Dillon, 2004; Bahia et al., 2014). Particularly, genomic, physiological and systematic characterization of commensal bacteria such as *S. marcescens* isolated from mosquitoes has been understudied (Pei et al., 2015). However, the study on bacterial interspecies competition demonstrated that *S. marcescens* could inhibited the growth of *Sphingomonas* and *Burkholderiaceae* members (Labbate et al., 2007). Competitive colonization was previously reported in the desert locust *Schistocerca gregaria* where bacterial diversity was shown to increase in the absence of *S. marcescens* (Dillon and Charnley, 2002). *In vitro* studies showed that extracts from *E. meningoseptica* had the antimicrobial properties, actively repressing gram-positive and negative bacteria and yeast (Ngwa et al., 2013). Our previous studies also showed *E. anophelis* survived in and consecutively associated with *A. stephensi* and *A. gambiae* mosquitoes rather than *A. triseriatus* (Chen et al., 2015). Further, we demonstrated that *E. anophelis* was more resistant to the digestion by *A. stephensi* and *A. gambiae* mosquitoes than by *A. triseriatus*, indicating that the bacterial ability to adapt in the gut environment is critical for persistence (Chen et al., 2015). Poor persistence of *S. fonticola* MSU001 in *A. stephensi* remains unexplored but it may be explained by its intolerance of the stress in the new host gut. Additional analyses are warranted to better understand the degree of interactions among the gut microbiota.

Mosquito-associated *S. marcescens* show distinctly different features from those isolated from environmental (free-living) or clinical settings (Lauzon et al., 2003; Iguchi et al., 2014; Lephoto and Gray, 2015). For example, our findings show that *S. marcescens* ano1 and ano2 lack genes encoding prodigiosins which are typically produced by many *Serratia* spp. and *Enterobacter* spp. (Williamson et al., 2006). Prodigiosins induced fragmentation of DNA, causing apoptosis in infected cells (Montaner et al., 2000). Prodigiosins were toxic to *P. falciparum* (Lazaro et al., 2002) and *T. cruzi* (Azambuja et al., 2004) and lethal to host mosquitoes (Patil et al., 2011; Suryawanshi et al., 2015). Absence of prodigiosins in mosquito-associated *Serratia* is consistent with their commensal life style. Moreover, another remarkable characteristic of commensal *S. marcescens* ano1 is production of urease with a complete urease gene cassette. By contrast, clinically important *Serratia* isolates are typically urease-negative. Urease, a nickel-containing metalloenzyme, catalyzes the hydrolysis reaction of urea and converts it to carbon dioxide and ammonia (Burne and Chen, 2000). In the mosquito or nematode native habitats (e.g., rice fields or soils), a high concentration of urea occurs after nitrogen fertilization (Victor and Reuben, 2000). Further, urease-producing, mosquito-associated *Serratia* possibly utilize available urea and arginine released from ingested animal erythrocytes as the nitrogen source for growth; consequently, the metabolites (such as ammonia) may contribute to regulate pH in the mosquito gut (Linser et al., 2009). On the other hand, some microbial ureases have insecticidal or fungal-toxic activity (Stanisçuaski et al., 2005; Becker-Ritt et al., 2007). This mode of toxicity relies on an internal peptide released upon proteolysis of ingested urease by insect digestive enzymes (Stanisçuaski et al., 2005). The intact protein and its derived peptide(s) are neurotoxic to insects or eukaryotic parasites and affect a number of other physiological functions such as diuresis, muscle contraction and immunity status (Stanisçuaski et al., 2005). Microbial ureases are fungitoxic to filamentous fungi and yeasts through a mechanism involving cell membrane permeabilization (Becker-Ritt et al., 2007).

*Serratia marcescens* ano1 has a sophisticated chitin digestion system that may facilitate depolymerization of chitin ingested with detritus by larval mosquitoes. Thus, *Serratia* may directly obtain nutrients from chitin degradation products, which may provide a labile carbon source to mosquito hosts (Vaaje-Kolstad et al., 2013). Furthermore, addition of chitotriose (intermediate chitin degradation products) into blood meal completely abolished *P. vivax* infectivity in *Anopheles tessellatus*. Chitotriose blocked the ookinete binding sites (GlcNAc residues) present in glycoproteins of the epithelium in gut. Moreover, chitinase secreted by *S. marcescens* could degrade the chitin structure on the gut lumen surface of insects (Hamilton et al., 2014), destroying the parasites' binding sites. However, more studies are warranted to elucidate if *S. marcescens* blocks malaria parasites' infection in mosquitoes through the chitinase production pathway.

*Serratia marcescens* secreted hemolysin which lysed animal erythrocytes in the blood meal; this facilitated acquisition of nutrients from the blood meal for the bacterial commensals and the mosquito host simultaneously (Gaio Ade et al., 2011). Removal of *Serratia* and/or other commensal bacteria caused reduced egg production, indicating that *Serratia* contributes to mosquito fecundity (Azambuja et al., 2004; Gaio Ade et al., 2011). Hemolysins in *Serratia* or *Serratia*-like bacteria had some different characteristics from the typical ones described previously (Ralf, 2005; Peraro and van der Goot, 2016). The hemolysin ShlA (OHT39701.1) in *S. marcescens* was secreted through and next activated by ShlB (OHT39702.1) which was a component of the two partner secretion system (TPSS, type V-secretion system) (Pramanik et al., 2014). After being processed, active format of secreted ShlA bond to erythrocytes and led to cell lysis by pore formation (Di Venanzio et al., 2014; Pramanik et al., 2014). In *S. marcescens*, genes *shlA* and *shlB* were tandemly organized and transcriptionally regulated by RcsB (Di Venanzio et al., 2014). The expression of *shlA* was induced under the low iron condition and low temperature. Furthermore, ShlA was also shown to have additional functions, such as promoting vacuolization and apoptosis in host. Besides blood cells, hemolysins damage epithelial barriers, thus promoting bacterial invasion and dissemination (Nagamatsu et al., 2015; Ristow and Welch, 2016). Direct contact between *S. marcescens* Db11 and infected *C. elegans* was required for killing effect, indicating that the function of hemolysins synergized other components in *S. marcescens* Db11 for pathogenicity (Kurz and Ewbank, 2000; Kurz et al., 2003). Moreover, hemolysins attacked various immune cells as an immune evasion strategy (Smith et al., 2015).

Serralysins, belonging to a conserved metalloprotease superfamily, degrade various host defense proteins such as immunoglobulins, antimicrobial peptides (defensin), complement proteins, as well as some structural proteins with barrier functions (Potempa and Pike, 2009; Ishii et al., 2014). *Serratia marcescens* PIC3611 had at least 4 experimentally-verified serralysins (i.e., PrtS, SlpB, SlpC, and SlpD; Shanks et al., 2015). Expression of *prtS* alone was sufficient for cytotoxicity of a corneal cell line (Shanks et al., 2015). However, various serralysins may have differential cytotoxicity, depending on the host cell type(s) (Ishii et al., 2014; Shanks et al., 2015). Serralysins in entomopathogenic *S. marcescens* notably increased the release of phagocytic hemocytes into silkworm hemolymph but it was not lethal to the moths (Ishii et al., 2014). Hemolymph invasion of *S. marcescens* Ss1 suppressed the immune response in honey bees, which led to fast bacterial growth in the bees (Burritt et al., 2016). The bacterial propagation in the hemolymph possibly caused permeabilization of organ membranes, resulting in leakage of circulatory and digestive contents into the hemolymph (Burritt et al., 2016).

Microbiota in the female mosquito may encounter two distinct iron-stressing circumstances, depending on the available food types (i.e., sugar or blood meal). When plant nectar is ingested, iron is very limited in the mosquito gut (Gonzales and Hansen, 2016). A sudden, high concentration of iron/heme will be available in the midgut when erythrocytes are ingested and digested by female mosquitoes (Benoit et al., 2011). Under the first scenario, gut microbiota need to scavenge iron either from the insect host or other microbes. *Serratia marcescens* may secrete enterobactin to acquire iron (Angerer et al., 1992). Collectively, our discovery of the heme uptake, transportation and storage gene clusters agree with previous reports of iron acquisition by *S. marcescens* (Angerer et al., 1992) and with the consistent presence and persistence of *Serratia* in mosquitoes. Enterobactin chelates a very low concentration of environmental ferric ion (Fe^3+^) at extremely high affinity (greater than EDTA) and delivers the soluble iron to the bacteria. Chelated and/or free forms of iron from the environment can be transported into bacterial cells through different iron transportation systems. It is very important for *Serratia* to produce siderophores with high iron affinity and have diverse iron transporters, which allows them to compete other microbiota under very limited iron in mosquito gut. On the other hand, the free radicals and oxidative molecules caused by high titers of heme or iron are harmful for some midgut bacteria after blood cells being lysed (Graça-Souza et al., 2006). Bacteria surviving in such environments are expected to have a capability to deal with these high iron stressing condition (Graça-Souza et al., 2006).

## Author contributions

SC and EW conceived the study and participated in its design and coordination. SC performed the experiments, whole genome sequencing, annotation, and comparative analysis. JB contributed to the genome analysis. SC and EW wrote the manuscript. All authors have read and approved the manuscript.

### Conflict of interest statement

The authors declare that the research was conducted in the absence of any commercial or financial relationships that could be construed as a potential conflict of interest.
