# Supplementary material for: Genomic, Physiologic, and Symbiotic Characterization of Serratia marcescens Strains Isolated from the Mosquito Anopheles stephensi
Source: Front Microbiol. 2017 Aug 10;8:1483. doi: 10.3389/fmicb.2017.01483 (PMC5561391; doi:10.3389/fmicb.2017.01483)
Supplement: Supplementary file 1 [file DataSheet1.DOCX]

**Table S1. Prophages predicted by PHAST in the selected *Serratia***

| **Strain** | **Region** | **Region length (kb)** | **Region position** | **Possible phage** | **GC content** | **Completeness** |
| --- | --- | --- | --- | --- | --- | --- |
| *S. marcescens* *ano1* | 1 | 6.2 | 289838-296135 | PHAGE_Entero_phi92_NC_023693 | 48.46% | incomplete |
|  | 2 | 7.6 | 536842-544465 | PHAGE_Escher_Av_05_NC_025830 | 61.28% | incomplete |
|  | 3 | 38.3 | 452500-490804 | PHAGE_Salmon_SEN5_NC_028701 | 57.22% | intact |
|  | 4 | 76.1 | 153588-229715 | PHAGE_Salmon_ST64B_NC_004313 | 54.42% | intact |
|  | 5 | 13.4 | 218329-231816 | PHAGE_Klebsi_phiKO2_NC_005857 | 56.95% | questionable |
| *S. marcescens* *ano2* | 1 | 6.2 | 289838-296135 | PHAGE_Entero_phi92_NC_023693 | 48.46% | incomplete |
|  | 2 | 7.6 | 536842-544465 | PHAGE_Escher_Av_05_NC_025830 | 61.28% | incomplete |
|  | 3 | 38.3 | 452500-490804 | PHAGE_Salmon_SEN5_NC_028701 | 57.22% | intact |
|  | 4 | 76.1 | 153588-229715 | PHAGE_Salmon_ST64B_NC_004313 | 54.42% | intact |
|  | 5 | 13.4 | 218329-231816 | PHAGE_Klebsi_phiKO2_NC_005857 | 56.95% | questionable |
| *S. marcescens* Db11 | 1 | 42.9 | 2744949-2787894 | PHAGE_Entero_SfV_NC_003444 | 54.96 % | intact |
| *S. marcescens* FGI94 | 1 | 17 | 878066-895075 | PHAGE_Yersin_413C | 55.47% | incomplete |
| *S. marcescens* WW4 | 1 | 14.1 | 99433-113628 | PHAGE_Entero_P4_NC_001609 | 55.95% | incomplete |
|  | 2 | 25 | 1003498-1028582 | PHAGE_Entero_P4_NC_001609 | 48.99% | incomplete |
|  | 3 | 34.9 | 2103728-2138659 | PHAGE_Haemop_Aaphi23_NC_004827 | 55.32% | intact |
|  | 4 | 16.7 | 2882093-2898874 | PHAGE_Entero_phiFL3A_NC_013648 | 51.43% | incomplete |
|  | 5 | 24.2 | 4584219-4608490 | PHAGE_Yersin_L_413C_NC_004745 | 56.93% | questionable |
| *S. plymuthica* S13 | 1 | 17.5 | 854555-872087 | PHAGE_Entero_P2 | 51.54% | incomplete |
|  | 2 | 7.9 | 1690698-1698647 | PHAGE_Plankt_PaV_LD | 49.85% | incomplete |
|  | 3 | 18.3 | 4902333-4920712 | PHAGE_Entero_N15 | 50.94% | incomplete |
| *S. proteamaculans* 568 | 1 | 26.4 | 398922-425399 | PHAGE_Entero_fiAA91_ss_NC_022750 | 53.02% | incomplete |
|  | 2 | 21.9 | 952334-974314 | PHAGE_Entero_P2_NC_001895 | 54.64% | incomplete |
|  | 3 | 23.4 | 2219775-2243202 | PHAGE_Vibrio_vB_VpaM_MAR_NC_019722 | 46.60% | questionable |
|  | 4 | 12.9 | 2464528-2477517 | PHAGE_Stx2_converting_I_NC_003525 | 50.52% | incomplete |
|  | 5 | 28.9 | 4518035-4546995 | PHAGE_Cafete_BV_PW1_NC_014637 | 53.94% | incomplete |
| *Serratia* sp. TEL | 1 | 8.3 | 529800-538114 | PHAGE_Campyl_CPt10_NC_027996 | 59.33% | incomplete |
| *S. fonticola* GS2 | 1 | 37 | 805654-842699 | PHAGE_Vibrio_8_NC_022747 | 53.63% | intact |
|  | 2 | 32 | 1526479-1558565 | PHAGE_Escher_HK75_NC_016160 | 53.36% | intact |
|  | 3 | 25.2 | 2276832-2302037 | PHAGE_Aeromo_vB_AsaM_56_NC_019527 | 49.81% | incomplete |
|  | 4 | 35.9 | 3131879-3167780 | PHAGE_Salmon_RE_2010_NC_019488 | 50.95% | intact |
|  | 5 | 55.7 | 3353638-3409424 | PHAGE_Edward_FW_3_NC_026611 | 51.27% | intact |
|  | 6 | 45.7 | 4808482-4854225 | PHAGE_Entero_IME10_NC_019501 | 51.80% | intact |
|  | 7 | 11.4 | 5195179-5206580 | PHAGE_Entero_P4_NC_001609 | 47.71% | incomplete |
|  | 8 | 17.3 | 5213314-5230619 | PHAGE_Cronob_phiES15_NC_018454 | 52.62% | questionable |
| *S. marcescens* MCB | 1 | 11.8 | 1105-12986 | PHAGE_Cronob_ENT39118_NC_019934 | 50.40% | incomplete |
|  | 2 | 9.2 | 216363-225564 | PHAGE_Plankt_PaV_LD_NC_016564 | 62.75% | incomplete |
|  | 3 | 11.4 | 288113-299604 | PHAGE_Parame_bursaria_Chlorella_virus_1_NC_000852 | 60.80% | incomplete |
|  | 4 | 9.8 | 120139-130009 | PHAGE_Rhodov_RS1_NC_020866 | 60.54% | incomplete |
| *S. liquefaciens* ATCC 27592 | 1 | 11.4 | 470114-481538 | PHAGE_Entero_P4_NC_001609 | 47.26% | incomplete |
|  | 2 | 42 | 1858200-1900259 | PHAGE_Salmon_ST160_NC_014900 | 54.63% | intact |
|  | 3 | 25.2 | 3008370-3033625 | PHAGE_Edward_FW_3_NC_026611 | 49.32% | incomplete |
|  | 4 | 29.9 | 3959409-3989354 | PHAGE_Klebsi_phiKO2_NC_005857 | 50.90% | intact |
| *Serratia* sp. Ag 1 | 1 | 7.1 | 78890-86024 | PHAGE_Plankt_PaV_LD_NC_016564 | 53.38% | incomplete |
|  | 2 | 3.5 | 18324-21852 | PHAGE_Entero_P4_NC_001609 | 46.27% | incomplete |
| *Serratia* sp. Ag 2 | 1 | 7.1 | 78887-86009 | PHAGE_Plankt_PaV_LD_NC_016564 | 53.39% | incomplete |
|  | 2 | 11.7 | 192947-204688 | PHAGE_Ectoca_siliculosus_virus_1_NC_002687 | 56.48% | incomplete |
|  | 3 | 39.1 | 13423-52533 | PHAGE_Shigel_SfIV_NC_022749 | 52.69% | questionable |
|  | 4 | 24.9 | 23-24947 | PHAGE_Entero_SfV_NC_003444 | 53.17% | intact |
| *S. marcescens* FS14 | 1 | 7 | 2101253-2108349 | PHAGE_Bacill_Grass_NC_022771 | 60.14% | incomplete |
|  | 2 | 5.3 | 2201184-2206502 | PHAGE_Chryso_virus_NC_028094 | 60.54% | incomplete |
|  | 3 | 26.4 | 2963533-2989944 | PHAGE_Staphy_SPbeta_like_NC_029119 | 50.17% | questionable |
|  | 4 | 12.6 | 3256468-3269071 | PHAGE_Bacill_G_NC_023719 | 59.63% | incomplete |
|  | 5 | 11.3 | 3324498-3335826 | PHAGE_Cafete_BV_PW1_NC_014637 | 53.91% | incomplete |
|  | 6 | 8.3 | 3901683-3909993 | PHAGE_Campyl_CPt10_NC_027996 | 58.77% | incomplete |
|  | 7 | 8.2 | 4396159-4404399 | PHAGE_Rhodov_RS1_NC_020866 | 59.85% | incomplete |

**Table S2. Antibiotic genes in *S. marcescens***

|  | **Locus ID** | | **Gene** | | **Functions** | | **Selected Serratia sp.** | | | | | | | | | | | | | | | | | | | | | |  |
| --- | --- | --- | --- | --- | --- | --- | --- | --- | --- | --- | --- | --- | --- | --- | --- | --- | --- | --- | --- | --- | --- | --- | --- | --- | --- | --- | --- | --- | --- |
|  |  | |  | |  | | **Mosquitoes** | | | | | | | | | **Fly** | | **Nematodes** | | | **Fungi** | | **Plants Milk** | | | **Paper** | |  |  |
|  |  | |  | |  | | **Ano1** | | **Ano2** | | **Ag1** | | **Ag2** | | **MCB** | | **Db11** | | **TEL** | **FGI94** | | **Sp** | | **Sl** | **WW4** | |  |  |  |
| **Efflux Pump** |  | |  | |  | |  | |  | |  | |  | |  | |  | |  |  | |  | |  |  | |  |  |  |
|  | OHT34382 | | *acrB* | | multidrug efflux RND transporter permease | | + | | + | | + | | + | | + | | + | | + | + | | + | | + | + | |  |  |  |
|  | OHT38912 | | *acrB* | | aminoglycoside/multidrug transporter permease | | + | | + | | + | | + | | + | | + | | + | + | | + | | + | + | |  |  |  |
|  | OHT38911 | | *acrA* | | efflux transporter periplasmic adaptor subunit | | + | | + | | + | | + | | + | | + | | + | + | | + | | + | + | |  |  |  |
|  | OHT33598 | | *tolC* | | outer membrane channel protein TolC | | + | | + | | + | | + | | + | | + | | + | + | | + | | + | + | |  |  |  |
|  | OHT35395 | | *oqxB* | | multidrug efflux RND transporter permease subunit | | + | | + | | - | | - | | + | | + | | + | - | | + | | + | + | |  |  |  |
|  | OHT37756 | | *mdfA* | | multidrug transporter MdfA | | + | | + | | + | | + | | + | | + | | + | - | | + | | + | + | |  |  |  |
|  | OHT36951 | | *mdtH* | | multidrug resistance protein MdtH | | + | | + | | + | | + | | + | | + | | - | - | | + | | + | + | |  |  |  |
|  | OHT35870 | | *oqxA* | | efflux transporter periplasmic adaptor subunit | | + | | + | | - | | - | | + | | + | | + | - | | + | | + | + | |  |  |  |
|  | OHT41355 | | *emrD* | | multidrug transporter EmrD | | + | | + | | - | | - | | + | | + | | + | + | | + | | + | + | |  |  |  |
|  | OHT38187 | | *mdtG* | | multidrug transporter subunit MdtG | | + | | + | | - | | - | | + | | + | | + | + | | + | | + | + | |  |  |  |
|  | OHT36431 | | *bcr-1* | | Bcr/CflA family drug resistance efflux transporter | | + | | + | | + | | + | | + | | + | | + | + | | + | | + | + | |  |  |  |
|  | OHT39857 | | *mdtD* | | multidrug transporter subunit MdtD | | + | | + | | + | | + | | + | | + | | + | - | | + | | + | + | |  |  |  |
|  | OHT39860 | | *mdtA* | | multidrug transporter subunit MdtA | | + | | + | | + | | + | | + | | + | | + | + | | + | | + | + | |  |  |  |
|  | OHT39858 | | *mdtC* | | multidrug transporter subunit MdtC | | + | | + | | + | | + | | + | | + | | + | + | | + | | + | + | |  |  |  |
|  | OHT39859 | | *mdtB* | | multidrug transporter subunit MdtB | | + | | + | | + | | + | | + | | + | | + | + | | + | | + | + | |  |  |  |
|  | OHT39856 | | *baeS* | | two-component system sensor histidine kinase BaeA | | + | | + | | + | | + | | + | | + | | + | + | | + | | + | + | |  |  |  |
|  | OHT39855 | | *baeR* | | two-component system response regulator BaeR | | + | | + | | + | | + | | + | | + | | + | + | | + | | + | + | |  |  |  |
|  | OHT41366 | | *cpxA* | | two-component system sensor histidine kinase CpxA | | + | | + | | + | | + | | + | | + | | + | + | | + | | + | + | |  |  |  |
|  | OHT41365 | | *cpxR* | | DNA-binding response regulator | | + | | + | | + | | + | | + | | + | | + | + | | + | | + | + | |  |  |  |
|  | OHT37058 | | *acrD* | | aminoglycoside/multidrug transporter subunit AcrD | | + | | + | | + | | + | | + | | + | | + | + | | + | | + | + | |  |  |  |
|  | OHT41510 | | *emrA* | | multidrug export protein EmrA | | + | | + | | + | | + | | + | | + | | + | + | | + | | + | + | |  |  |  |
|  | OHT36624 | | *mdtK* | | FMN/FAD transporter | | + | | + | | + | | + | | + | | + | | + | + | | + | | + | + | |  |  |  |
|  | OHT38246 | | *mexH* | | efflux transporter periplasmic adaptor subunit | | + | | + | | - | | - | | + | | + | | - | - | | - | | - | + | |  |  |  |
|  | OHT38245 | | *mexI* | | multidrug efflux protein | | + | | + | | - | | - | | + | | + | | - | - | | - | | - | + | |  |  |  |
|  | OHT41509 | | *emrB* | | multidrug resistance protein B | | + | | + | | + | | + | | + | | + | | + | + | | + | | + | + | |  |  |  |
|  | OHT40492 | | *macA* | | efflux transporter periplasmic adaptor subunit | | + | | + | | + | | + | | - | | - | | + | + | | + | | - | - | |  |  |  |
|  | OHT38334 | | *macB* | | macrolide ABC transporter permease/ATP-binding protein MacB | | + | | + | | + | | + | | + | | + | | + | + | | + | | + | + | |  |  |  |
|  | OHT38335 | | *macA* | | macrolide transporter subunit MacA | | + | | + | | + | | + | | + | | + | | + | + | | + | | + | + | |  |  |  |
|  | OHT40393 | | *macB* | | macrolide ABC transporter permease/ATP-binding protein MacB | | + | | + | | + | | + | | - | | - | | + | + | | + | | - | - | |  |  |  |
|  | OHT38205 | | *macB* | | macrolide ABC transporter permease/ATP-binding protein MacB | | + | | + | | - | | - | | + | | + | | + | - | | + | | + | + | |  |  |  |
|  | OHT38968 | | *macA* | | efflux transporter periplasmic adaptor subunit | | + | | + | | - | | - | | + | | + | | + | - | | + | | + | + | |  |  |  |
|  | OHT37642 | | *CRP* | | transcriptional regulator Crp | | + | | + | | + | | + | | + | | + | | + | + | | + | | + | + | |  |  |  |
|  | OHT38893 | | *rosB* | | Kef family K(+) transporter | | + | | + | | + | | + | | + | | + | | + | + | | + | | + | + | |  |  |  |
|  | OHT38892 | | *rosA* | | Fosmidomycin resistance protein | | + | | + | | + | | + | | + | | + | | + | + | | + | | + | + | |  |  |  |
|  | OHT34360 | | *tet41* | | tetracycline resistance MFS efflux pump | | + | | + | | - | | - | | + | | + | | + | - | | - | | - | + | |  |  |  |
|  | OHT32222 | | *GlpT* | | transcriptional regulator, *fosfomycin* | | + | | + | | + | | + | | + | | + | | - | + | | + | | + | + | |  |  |  |
|  | OHT34920 | | *leuO* | | transcriptional regulator LeuO | | + | | + | | + | | + | | + | | + | | + | + | | + | | + | + | |  |  |  |
| **Antibiotics resistance genes** | | | | | | | | | | | | | | | | | | | | | | | | | | | | | |
| **fosfomycin** | | | | | | | | | | | | | | | | | | | | | | | | | | | | | |
|  | | OHT33384 | | *gpt* | | glycerol-3-phosphate transporter | | + | | + | | + | | + | + | | + | | + | + | | + | | + | + | |  |  |  |
| **mupirocin** | | | | | | | | | | | | | | | | | | | | | | | | | | | | | |
|  | | OHT34958 | | *ileS* | | isoleucine--tRNA ligase, mupirocin | | + | | + | | + | | + | + | | + | | + | + | | + | | + | + | |  |  |  |
| **polymyxin** | | | | | | | | | | | | | | | | | | | | | | | | | | | | | |
|  | | OHT35737 | | *PmrC* | | phosphoethanolamine transferase EptA | | + | | + | | + | | + | + | | + | | + | + | | + | | + | + | |  |  |  |
|  | | OHT42333 | | *PmrB* | | two-component system sensor histidine kinase BasS | | + | | + | | + | | + | + | | + | | + | + | | + | | + | + | |  |  |  |
|  | | OHT35690 | | *PmrF* | | undecaprenyl-phosphate 4-deoxy-4-formamido-L-arabinose transferase | | + | | + | | + | | + | + | | + | | + | + | | + | | + | + | |  |  |  |
|  | | OHT35896 | | *arnA* | | ifunctional UDP-glucuronic acid oxidase/UDP-4-amino-4-deoxy-L-arabinose formyltransferase | | + | | + | | + | | + | + | | + | | + | + | | + | | + | + | |  |  |  |
| **aminocoumar** | | | | | | | | | | | | | | | | | | | | | | | | | | | | | |
|  | | OHT34833 | | *alaS* | | aminocoumarin resistant gene, alanine--tRNA ligase | | + | | + | | + | | + | + | | + | | + | + | | + | | + | + | |  |  |  |
|  | | OHT32273 | | *cysB* | | aminocoumarin resistant transcriptional regulator CysB | | + | | + | | + | | + | + | | + | | + | + | | + | | + | + | |  |  |  |
|  | | OHT38790 | | *kdpE* | | two-component system response regulator KdpE | | + | | + | | + | | + | + | | + | | + | + | | + | | + | + | |  |  |  |
|  | | OHT33574 | | *AAC(6')-Ic* | | aminoglycoside N-acetyltransferase AAC(6')-Ic | | + | | + | | - | | - | + | | + | | + | - | | - | | - | + | |  |  |  |
| **beta-lactam** | | | | | | | | | | | | | | | | | | | | | | | | | | | | | |
|  | | OHT35785 | | *SRT-2* | | class C beta-lactamase | | + | | + | | - | | - | + | | + | | + | + | | + | | + | + | |  |  |  |
|  | | OHT35784 | | *NmcR* | | LysR family transcriptional regulator | | + | | + | | - | | - | + | | + | | + | - | | + | | + | + | |  |  |  |
|  | | OHT33815 | | *omp36* | | porin OmpC | | + | | + | | - | | - | + | | + | | + | + | | + | | + | + | |  |  |  |
| **isoniazid** | | | | | | | | | | | | | | | | | | | | | | | | | | | | | |
|  | | OHT36528 | | *katG* | | catalase/peroxidase HPI | | + | | + | | - | | - | + | | + | | + | + | | + | | + | + | |  |  |  |
| **fluoroquinolone** | | | | | | | | | | | | | | | | | | | | | | | | | | | | | |
|  | | OHT35459 | | *Mfd* | | transcription-repair coupling factor | | + | | + | | + | | + | + | | + | | + | + | | + | | + | + | |  |  |  |

* Abbreviations: Ano1, *S. marcescens* ano1; Ano2, *S. marcescens* ano2; Ag1, *Serratia* sp. Ag1; Ag2, *Serratia* sp. Ag2; MCB, *S. marcescens* MCB; Db11, *S. marcescens* Db11; TEL, *Serratia* sp. TEL; FGI94, *S. marcescens* FGI94; Sp, *S. plymuthica* S13; Sl, *S. liquefaciens* ATCC 27592; WW4, *S. marcescens* WW4.

**Table S3. Antimicrobial compounds producing in selected *Serratia* spp.**

| Antimicrobials | Ano 1 | Ano 2 | Db11 | FGI94 | WW4 | S13 | Sp568 | TEL | MCB | Ag 1 | Ag 2 | FS14 | ATCC 27592 |
| --- | --- | --- | --- | --- | --- | --- | --- | --- | --- | --- | --- | --- | --- |
| Entericidin | + | + | + | + | + | + | + | + | + | - | - | + | + |
| Microcin C | - | + | + | - | + | + | - | + | + | + | + | + | + |
| Microcin H47 | - | - | - | - | + | - | - | + | + | - | - | + | - |
| S-type pyocin | - | - | - | - | - | - | + | - | - | - | - | + | - |
| Microcin H47 | + | + | + | - | + | + | - | + | - | - | - | + | + |
| S-type pyocin | + | + | - | - | + | + | + | - | + | + | + | + | + |
| Microcin H47 | - | - | - | - | - | - | - | - | - | - | - | - | - |
| bacteriocin | - | - | + | - | - | - | - | - | + | - | - | - | - |
| S-type pyocin | + | + | + | - | - | - | - | - | - | - | - | - | - |
|  |  |  |  |  |  |  |  |  |  |  |  |  |  |

* Abbreviations: Ano1, *S. marcescens* ano1; Ano2, *S. marcescens* ano2; Ag1, *Serratia* sp. Ag1; Ag2, *Serratia* sp. Ag2; MCB, *S. marcescens* MCB; Db11, *S. marcescens* Db11; TEL, *Serratia* sp. TEL; FGI94, *S. marcescens* FGI94; Sp, *S. plymuthica* S13; Sl, *S. liquefaciens* ATCC 27592; WW4, *S. marcescens* WW4.

**Figure S4. Comparisons of pigment production genes in the selected *Serratia***

| **Gene** | **Functions** | **ano1** | **ano2** | **Db11** | **FGI94** | **Sp** | **Sp568** | **TEL** | **MCB** | **Ag1** | **Ag2** | **Fs14** | **ATCC 27592** | **WW4** |
| --- | --- | --- | --- | --- | --- | --- | --- | --- | --- | --- | --- | --- | --- | --- |
| *cueR* | transcriptional regulator of CopA | + | + | + | + | + | + | + | + | + | + | + | + | + |
| *pigA* | acyl-CoA dehydrogenase | - | - | - | - | - | - | - | - | - | - | + | - | + |
| *pigB* | oxidoreductase | - | - | - | - | - | - | - | - | - | - | + | - | + |
| *pigC* | phosphoenolpyruvate-utilizing enzyme | - | - | - | - | - | - | - | - | - | - | + | - | + |
| *pigD* | carbon transferase | - | - | - | - | - | - | - | - | - | - | + | - | + |
| *pigE* | aminotransferase | - | - | - | - | - | - | - | - | - | - | + | - | + |
| *pigF* | O-methyl transferase | - | - | - | - | - | - | - | - | - | - | + | - | + |
| *pigG* | peptidyl carrier protein | - | - | - | - | - | - | - | - | - | - | + | - | + |
| *pigH* | aminotransferase | - | - | - | - | - | - | - | - | - | - | + | - | + |
| *pigI* | L-prolyl-AMP ligase | - | - | - | - | - | - | - | - | - | - | + | - | + |
| *pigJ* | Beta-ketomyristol-ACP synthase | - | - | - | - | - | - | - | - | - | - | + | - | + |
| *pigK* | not assigned | - | - | - | - | - | - | - | - | - | - | + | - | + |
| *pigL* | 4'-phosphopantetheinyl transferase | - | - | - | - | - | - | - | - | - | - | + | - | + |
| *pigM* | oxidoreductase | - | - | - | - | - | - | - | - | - | - | + | - | + |
| *pigN* | oxidoreductase | - | - | - | - | - | - | - | - | - | - | + | - | + |
| *copA* | copper transporting ATPase efflux pump | + | + | + | - | + | - | + | + | + | + | + | + | + |

* Abbreviations: Ano1, *S. marcescens* ano1; Ano2, *S. marcescens* ano2; Ag1, *Serratia* sp. Ag1; Ag2, *Serratia* sp. Ag2; MCB, *S. marcescens* MCB; Db11, *S. marcescens* Db11; TEL, *Serratia* sp. TEL; FGI94, *S. marcescens* FGI94; Sp, *S. plymuthica* S13; Sl, *S. liquefaciens* ATCC 27592; WW4, *S. marcescens* WW4.

**Figure S1. Subsystem category distribution of *S. marcescens* ano1 (A) and ano2 (B) based on RAST server-based SEED viewer.**

**
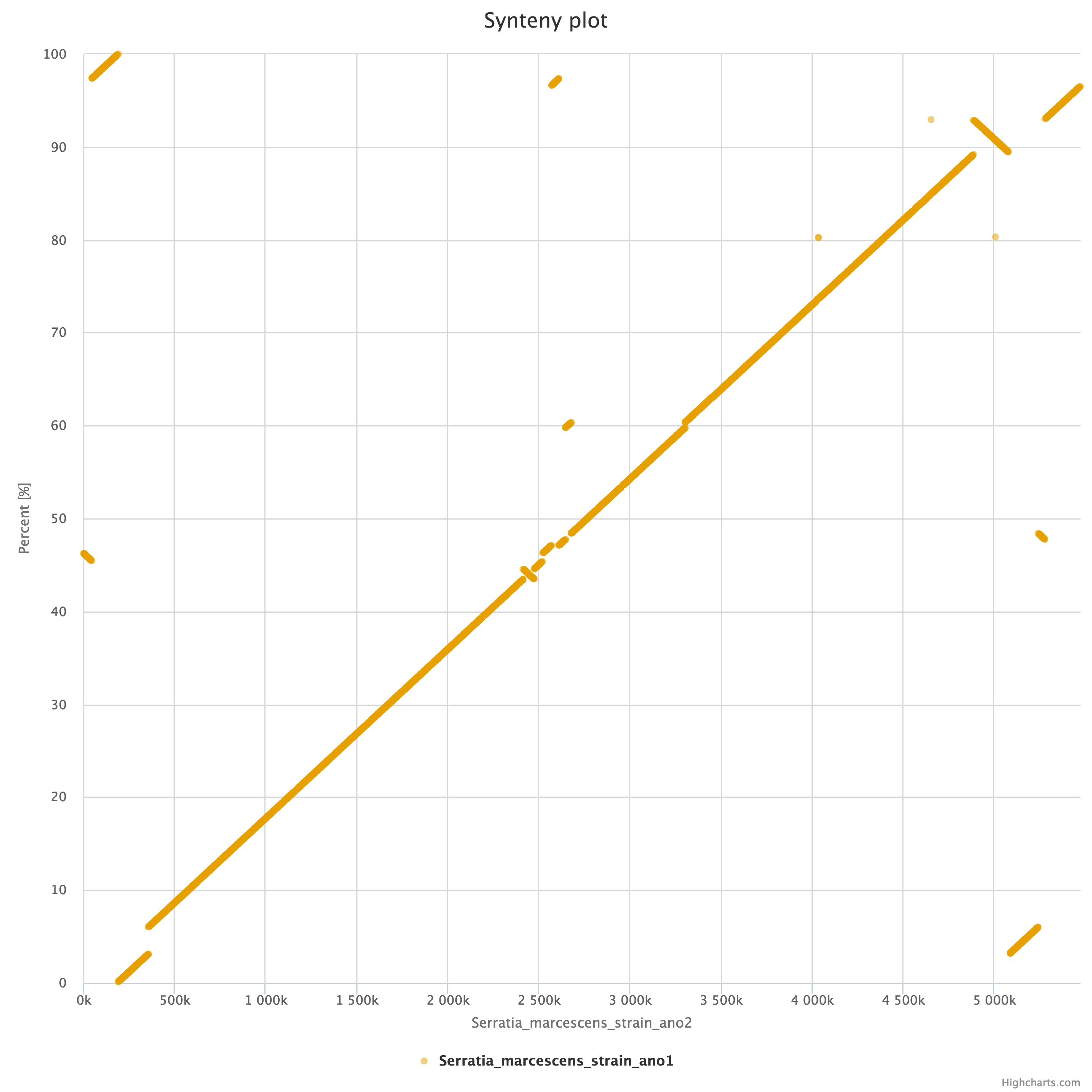
**

**Figure S2. Alignment analysis of *S. marcescens* ano1 and ano2.**

**Figure S3. Phylogenetic analysis of the *Serratia* spp.**


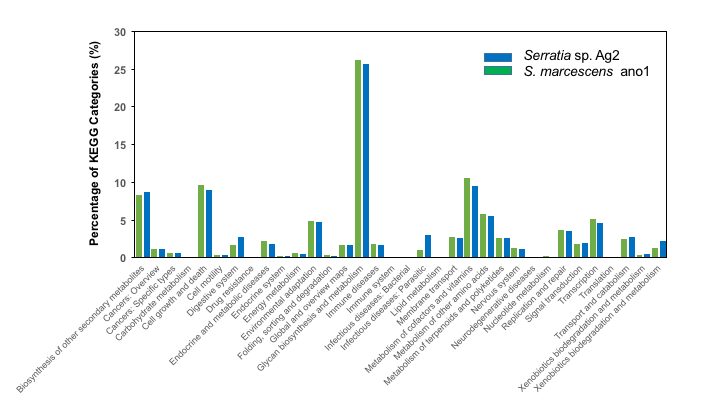


**Figure S4. Comparative KEGG categories analysis between two mosquito isolates *S. marcescens* ano1 and *Serratia* sp. Ag2**. *S. marcescens* ano1 was isolated from *A. stephensi* and *Serratia* sp. Ag2 was obtained from *A. gambiae*. The analysis of functional categories was conducted by using the KEGG server (http://www.genome.jp/kegg/ko.html).

**Figure S5. *Ure* gene clusters in three *Serratia* species.**
